# A study on the influence mechanism of CBDC on monetary policy: An analysis based on e-CNY

**DOI:** 10.1371/journal.pone.0268471

**Published:** 2022-07-08

**Authors:** Jiemeng Yang, Guangyou Zhou

**Affiliations:** School of Economics, Fudan University, Shanghai, China; URV: Universitat Rovira i Virgili, SPAIN

## Abstract

This paper attempts to introduce central bank digital currency (CBDC) into the analysis framework of monetary policy, and studies the influence mechanism of e-CNY, central bank digital currency in China, on the monetary policy of the central bank from the aspects of money demand, money supply and monetary policy transmission mechanism. The research finds that e-CNY will have significant impact on monetary policy: (1) E-CNY will change the structure of money demand, speed up currency circulation, make central bank reserves more controllable and money supply more intelligent; (2) E-CNY will increase the volatility and expansion effect of currency multiplier to a certain extent; (3) E-CNY will dredge the transmission channel of monetary policy so as to improve the transmission effect of existing monetary policy tools. At the same time, based on the organic combination with structural monetary policy tools, it will achieve precise implementation of medium-term lending facilities (MLF), pledged supplementary lending (PSL), and it may bring new monetary policy tools. (4) E-CNY will make the intermediate target of monetary policy more controllable and reliable, and have a positive impact on the target of monetary policy through the smooth transmission of monetary policy channels. Therefore, it is necessary to strengthen the research on CBDC, give full play to the positive role of e-CNY in monetary policy, and improve the effectiveness of monetary policy.

## 1. Introduction

Since the advent of Bitcoin in 2008, digital currencies have attracted extensive attention from scholars all over the world [1–7]. Digital currencies can be issued in two ways: decentralized and centralized [8]. Among them, most privately issued digital currencies such as Bitcoin belong to decentralized digital currencies, while central bank digital currencies (CBDCs) are centralized currencies issued by central banks, which are essentially different from each other. Once, many scholars focused on Bitcoin and its impact on the economy [9–14]. In recent years, a surging number of scholars have paid attention to the impact of central bank digital currency on the economy and many other aspects such as technology and law [15–17]. CBDC is the digital form of legal tender, which is endorsed by national credit. E-CNY is the digital version of fiat currency issued by the PBOC and operated by authorized operators. According to the latest White Paper of E-CNY, it is a value-based, quasi-account-based and account-based hybrid payment instrument, with legal tender status and loosely-coupled account linkage [18]. This paper mainly analyzes e-CNY, China’s central bank digital currency, and its impact on monetary policy.

In recent years, central banks of various countries attach great importance to the research of CBDC. According to the third survey released by the Bank for International Settlements (BIS) in 2021, 86% of economies are now participating in research on CBDCs, an increase of a third over the past four years, about 60% of central banks are conducting experiments or proof-of-concept (up from 42% in 2019), and 14% of central banks are advancing related trials [19]. China has been leading the world in CBDC research and development since 2014, when it set up a related group. Yao Qian, former director of the PBOC Digital Currency Research Institute, put forward the original idea and initial design of China’s CBDC [20, 21]. Since April 2020, China’s central bank digital currency project DC/EP (Digital Currency/Electronic Payment) has carried out closed internal pilot tests in Shenzhen, Suzhou, Xiong’an New Area and Chengdu. Subsequently, China’s central bank digital currency was officially named E-CNY. After that, various pilot activities of e-CNY are being carried out in full swing in twenty-three cities all over the country to gradually bring e-CNY into people’s lives. The pilot cities include Shenzhen, Suzhou, Xiong’an and Chengdu (first batch, declared as a pilot city at the end of 2019, and begins to pilot at April 2020); Shanghai, Hainan, Changsha, Xi’an, Qingdao, Dalian and 2022 Beijing Winter Olympics scenes: Beijing and Zhangjiakou (second batch, begins in November 2020); Tianjin, Chongqing, Guangzhou, Guangdong, Fuzhou, Xiamen, and 6 cities(Hangzhou, Ningbo, Wenzhou, Jinhua, Shaoxing, Huzhou) in Zhejiang that host the Asian Games (third batch, begins in April 2022).According to the latest statistics, by the end of 2021, 260 million e-CNY personal wallets and 10 million public wallets have been opened, and the cumulative transaction amount of e-CNY is about 87.5 billion yuan.

As an emerging form of currency, the issuance and circulation mode of CBDC is essentially different from that of traditional paper currency and electronic currency. Compared with traditional currency, it has features and advantages such as stability, convenience, low cost, universality, programmability, liquidity, and security, and is becoming a trend of currency development. While it brings convenience to people, it also has an obvious impact on the economy, among which the impact on monetary policy is the first. Although the impact of digital currency on monetary policy has been widely concerned by the central banks, academia, and industry since its emergence [22–27], the relevant studies are not specific enough. The studies of most scholars are more at the level of qualitative policy analysis. Moreover, many studies cannot objectively reflect the actual situation of e-CNY issuance and circulation. Therefore, as the circulation of CBDC is about to expand, in-depth research of the impact of CBDC on monetary policy has important theoretical and practical significance for the central bank to regulate the amount of digital currency issued, flexibly use and innovate monetary policy tools, dredge the transmission channel of monetary policy, and then improve the effectiveness of monetary policy.

The innovation of this paper lies in: (1) The research perspective is novel. From the perspective of digital currency replacing traditional currency, this paper studies the substitution effect of e-CNY on traditional currency and its influence on monetary policy. (2) This paper proposes a theoretical analysis framework of monetary policy based on central bank digital currency. This paper introduces CBDC into the theoretical framework of monetary policy analysis, and deeply studies the influence of CBDC on money supply, money demand and monetary policy transmission mechanism. (3) Theoretical model construction. A theoretical model of money supply and money demand based on CBDC is constructed to describe the influence of digital currency on money supply and demand. (4) This paper analyzes the influence of CBDC on the transmission mechanism of monetary policy from the aspects of monetary policy tools, intermediate targets and ultimate targets.

The structure of this paper is as follows: The first part is introduction; The second part is literature review; The third part is the influence of e-CNY on currency demand; The fourth part is the influence of e-CNY on money supply; The fifth part is the impact of e-CNY on the transmission mechanism of monetary policy; The sixth part summarizes the full text and puts forward relevant policy suggestions.

## 2. Literature review

### 2.1 Research on the impact of e-money on monetary policy

Although the issuance and circulation modes of central bank digital currency and electronic currency are different, and their influences on financial economy are different, they are both substitutes for legal tender, which is ultimately manifested as electronic currency. Therefore, relevant literature should be traced back to electronic currency.

At present, the definition of electronic money(e-money) is still controversial, but the definition proposed by the Basel Committee on Banking Supervision (BCBS) (1998) [28] is more generally accepted. BCBS defined e-money as "stored value" or prepaid payment mechanisms for executing payments via point of sale terminals, direct transfers between two devices, or over open computer networks such as the Internet.

Many scholars have carried out many empirical studies on the impact of e-money on the traditional monetary system and monetary policy. Fälth [29] pointed out that OECD countries that have experienced e-commerce development for many years have put forward research on the impact of digital currency and electronic currency on money circulation speed and money multiplier at the end of last century. Marko [30] pointed out that the wide spread of e-money may greatly narrow the balance sheet of the central bank and reduce the amount of base money, which may have an adverse impact on the implementation of monetary policy. Skeie [31] pointed out that the bank’s internal money in the form of electronic money can maintain the stability of the whole system, but it has no substantive impact on monetary policy. Fujiki et al. [32] constructed a household money demand function for e-money by using Japanese micro-data and found that money demand and e-money adoption increased simultaneously.

### 2.2 The impact of central bank digital currency on the whole economy system

For the interpretation of relevant concepts of central bank digital currency, the most recognized definition is the currency issuance framework of "the money flower: a taxonomy of money " proposed by Bench and Garratt [8], which defines its four major attributes: widely accessible, digital, central bank-issued, and token-based. CMPI [33] defines central bank digital currency as a new variant of central bank currency, which is different from the currency in physical cash, bank reserve requirements or settlement accounts.

Many scholars studied the overall impact of central bank digital currency on the economy. Some of them think that the issuance of CBDC will have an overall positive impact. Sinelnikova-Muryleva [34] discussed the potential risks and benefits of CBDC as well as their consequences for the banking sector and monetary policy, and found that with proper design, it will become a new effective tool of monetary authorities. Cullen [35] considers the issuance of CBDC in the Eurozone and believes that it will be economically efficient and may impair the development of payments systems and obstruct financial inclusion. Wu and Chen [36] conducted a quantitative analysis of the economic impact of the issuance of DC/EP based on a four-sector DSGE model and found that the substitution effect of DC/EP on bank deposits is limited, while the unit impact can enhance the economic growth rate by 0.15% and the overall economic effect is positive. Lee, Yan and Wang [37] compared the CBDC designs over the globe and found that the suitable design of CBDC will allow central banks to manage the process flow, focus on the monitoring and control, without bearing all the load or exposing to over-centralized risks and it will be the primary tool in the future digital economy. Kochergin [38] also compared different types of CBDC over the world and concluded the advantages and motivations and summarized the reasons for different types of issues in different countries.

Some scholars also discuss CBDC’s potential risks and challenges. Kirkby [39] pointed out that central bank issuing digital fiat currency will replace cash and other forms of money broadly, but he also thought if the central bank is responsible for the entire money supply, it may have side effects. Bindseil [40] discusses two key arguments against CBDC, the risk of structural disintermediation of banks and risk of facilitation systemic runs on banks in crisis situations, he found that well-controlled CBDC seems feasible, but there will still be catalyze change in the financial system. Cukierman [41] considered two paths of CBDC and examined the impact on the funding of banks, the allocation of credit to the economy, welfare, and political feasibility. He pointed out that in the situation that only the banking sector can have access to deposits at the central bank, CBDC will have little impact, however, the radical implementation that allows all private sector accessible to deposits at the central bank, it may cause a narrow banking system and dramatically reduce the need for deposit insurance in the long run. Belke and Beretta [42] explored the precarious balance between modernizing monetary systems by means of digital currencies and safeguarding financial stability as also ensured by tangible payment instruments like paper money, and found that there are still high risks connected to the introduction of central bank digital currency, which should be by far not considered to be a perfect substitute for cash. Hansen and Delak [43] discussed the security consideration of CBDC and pointed out the potential attacks on CBDC systems and given a CBDC designs based on distributed ledger technology (DLT). Samudrala and Yerchuru [44] considered the business and technology risks and challenges associated with CBDC implementation from the prospect of credit growth, distributed ledger technology, payment modernization and customer experience, and tried to propose key design considerations for CBDC exploration in India.

### 2.3 The impact of central bank digital currency on the effectiveness of monetary policy

Some scholars believe that issuing CBDC will help to improve the effectiveness of monetary policy, and have reached consensus in this regard. Engert and Fung [45] explored the motivations and implications of central bank digital currency from the fields of central bank seigniorage, monetary policy, banking system, financial stability, and payment convenience. Pfister [46] believes that if the issuance of CBDC follows the existing rules of currency issuance, the role of banks in allocating credit will not be seriously changed. Meaning et al. [47] studied the various stages of transmission of CBDC from the central bank to the real economy, found that monetary policy would be able to operate much as it does now, by varying the price or quantity of central bank money, and that transmission may even strengthen for a given change in policy instruments. Brunnermeier and Niepelt [48] constructed a currency model that can identify liquidity bubbles and seigniorage, and conducted simulation analysis of the model results with Project Chicago cryptocurrency, India’s experiment of non-monetization and central bank digital currency, proving that CBDC does not undermine financial stability. Andolfatto [49] investigated the impact of CBDC on a monopolistic banking sector and found that the introduction of a central bank digital currency has no detrimental effect on lending activity and may even promote it. Davoodalhosseini [50] studied the optimal monetary policy when only cash, only a CBDC, or both cash and a CBDC are available to agents, and pointed out that if the cost of CBDC is acceptable, it is a more efficient allocation. Monnet et al. [51] argue that CBDC can lead to less bank risk taking and higher output and welfare. Barrdear and Kumhof [52] conducted a DSGE model with CBDC and estimated that the issuance of CBDC could increase GDP by as much as 3%.

However, some scholars have also raised concerns about central bank digital currencies on monetary policy. Broadbent [26] worried about the "narrow banking" phenomenon of commercial bank deposits migrating to the central bank on a large scale after the implementation of CBDC. Rahman [53] examined the implications of digital and fiat currency competition on optimal monetary policy and found that if digital currencies compete with government-issued fiat money, the Friedman rule will be socially inefficient. Bjerg [54] studied how CBDC co-exists and interacts with existing monetary forms, and evaluated three different schemes for implementing CBDC from the perspective of monetary policy impact, found that a monetary system consisting of two competing money creators -the central bank and the commercial bank- still has a trilemma of free convertibility between CBDC and bank money, parity between CBDC and bank money, and central bank monetary sovereignty. Raskin and Yermack [55] pointed out that CBDC may have a far-reaching impact on the banking system, close the relationship between the people and the central bank, and reduce the public’s demand for retaining deposits in some commercial banks. Bjerg and Nielsen [24] studied the prospect analysis of the implementation of the central bank digital currency in Denmark and believed that the CBDC would not increase the efficiency of the existing payment solutions. Keister and Sanches [56] argued that CBDC may also crowd out bank deposits, raise bank funding costs and reduce investment, although the introduction of CBDC will generally improve social welfare. Fernandez-Villaverde et al. [27] found that the introduction of CBDC allows the central bank to engage in large-scale intermediation by competing with private financial intermediaries for deposits while in the panic time, CBDC may attract deposits away from the commercial banking sector, which might endanger maturity transformation.

### 2.4 The impact of central bank digital currency on interest rate channels and monetary policy tools

As digital euro, digital Yen, e-Krona of Sweden and other countries’ central bank digital currencies carry interest at the beginning of design, some scholars focus on the impact of issuing CBDC on interest rate channels. Bordo and Levin [57] believe that a monetary policy framework with CBDC should be accompanied by interest, and this framework will promote price stability. Besides, the effective lower bound may even be eliminated together with cash. Norges Bank [58] believes that the impact of CBDC on bank lending rates and overall lending channels is not obvious. Sveriges Riksbank [59] found that if commercial banks finance from the central bank, the transmission from the central bank interest rate to the loan interest rate may be stronger. If banks prefer inter-bank financing, the central bank may not be able to affect the loan interest rate. Harrison and Thomas [60] considered CBDC with interest income and found that it can use money-financed transfers as a policy tool within the effective lower limit without giving up its ability to use short-term bond interest rates to stabilize the economy in normal times. However, Gurtler et al. [61] pointed out that the deposit outflow of commercial banks and the related needs to provide external funds from different sources caused by CBDC do pose challenges to financial stability and have a potential impact on the efficiency of the entire monetary transmission mechanism. Wadsworth [62] assessed the advantages and disadvantages of the public digital currency issued by the central bank in the four functional areas of currency distribution, payment, currency stability and financial stability, and the advantages and disadvantages of digital currency among different functions of the central bank need to be weighed. Yanagawa and Yamaoka [63] believe that the central bank should not only pay attention to the impact of digital currency on the payment system, but also the impact of the entire financial system, especially the impact of network externalities.

Some scholars think that the introduction of CBDC will bring new monetary policy tools. Sveriges Riksbank [59] believes that with the weakening of the role of cash, the effective lower bound may decline, because the central bank is easier to affect the interest rate in the economy by setting CBDC interest rate, so as to expand the space of monetary policy. Harrison and Thomas [60] found that they can use the transfer of monetary financing as a policy tool within the effective lower bound without giving up the ability to use short-term bond interest rates to stabilize the economy in normal times. Dong and Xiao [64] argued that cash and interest-bearing CBDC can coexist, where the coexistence may require the central bank to adjust either the CBDC interest rate or the interest rate on reserves, and they also pointed out that some forms of CBDC can help implementing a negative interest rate.

As for the influence of central bank digital currency on monetary policy, many scholars have conducted exploratory research, mainly focusing on interest-bearing CBDC, which is not in accordance with the non-interest-bearing characteristics of e-CNY that replacing M0. Therefore, this paper will analyze the impact of e-CNY on the central bank’s monetary policy from the perspective of mechanism, and analyze the impact of e-CNY on money demand, money supply and monetary policy transmission mechanism in combination with the characteristics of multiple rounds of pilot projects of e-CNY and the relevant patents applied by the Digital Currency Research Institute of the People’s Bank of China, in order to get some meaningful and enlightening conclusions, and provide a reference for the central bank to issue digital currency in the future.

## 3. The impact of e-CNY on money demand

**Fig 1:**
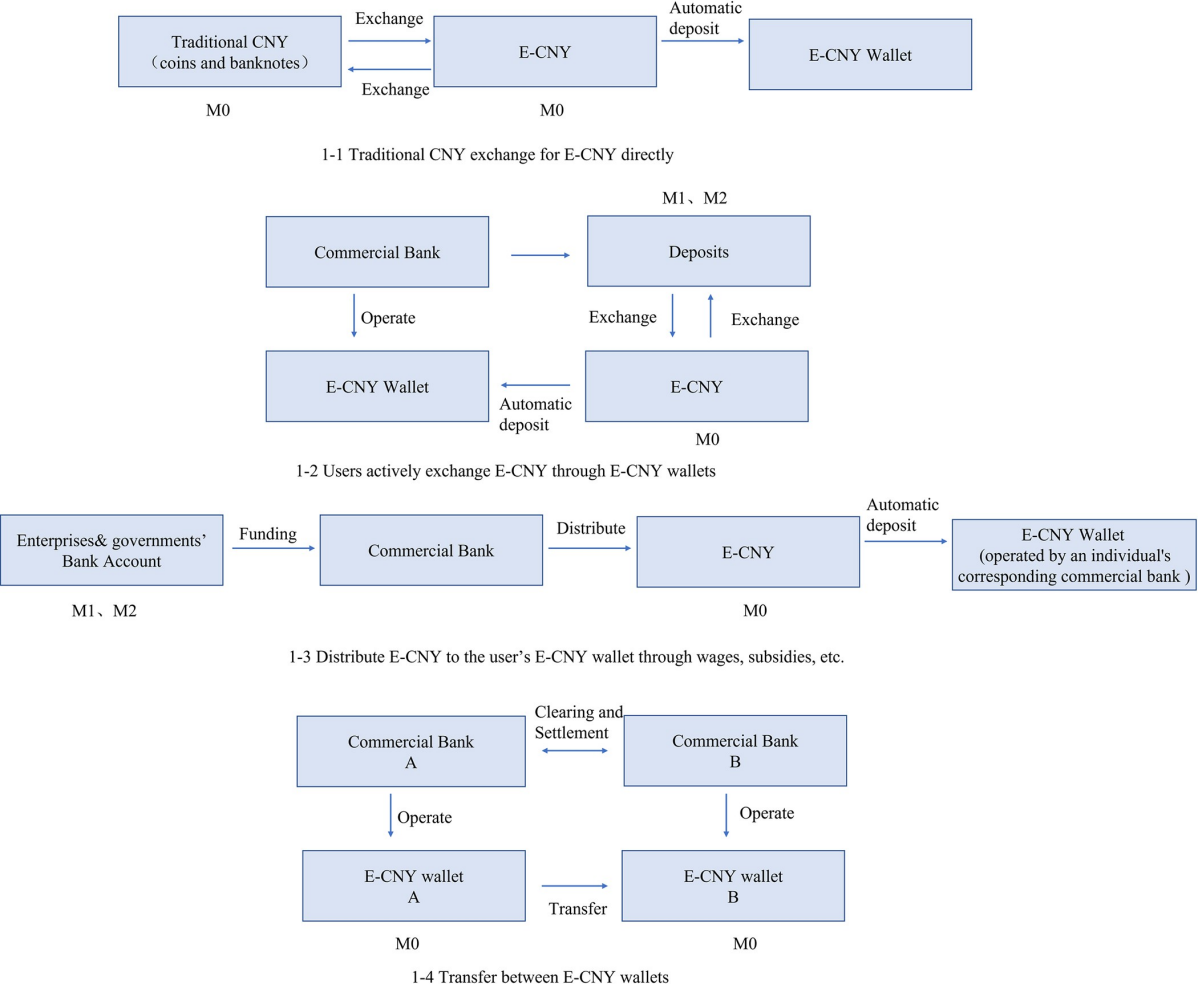
Four issuing and exchanging channels of e-CNY.

As defined in the *Progress of Research & Development of E-CNY in China* [18], e-CNY is mainly a substitute for cash in circulation (M0), which is the most liquid form of money. According to the current pilot situation, the issuance and exchange of e-CNY can be divided into four channels: First, users can directly exchange e-CNY with traditional CNY (including coins and banknotes, the following uses traditional CNY and e-CNY as a distinction). Second, users take the initiative to exchange e-CNY through the e-CNY wallet of commercial banks as operating institutions. Third, e-CNY is distributed to users’ e-CNY wallet through the channels of commercial banks by wages and red envelope subsidies, but the central bank does not directly distribute e-CNY to individuals because e-CNY adopts a two-tier operational system. Fourth, the transfer between e-CNY wallets. See Fig 1. These four channels of issuance and exchange will have different effects on China’s monetary structure.

### 3.1 E-CNY will replace most paper CNY, and the total demand for M0 will increase first and then decrease

**Fig 2:**
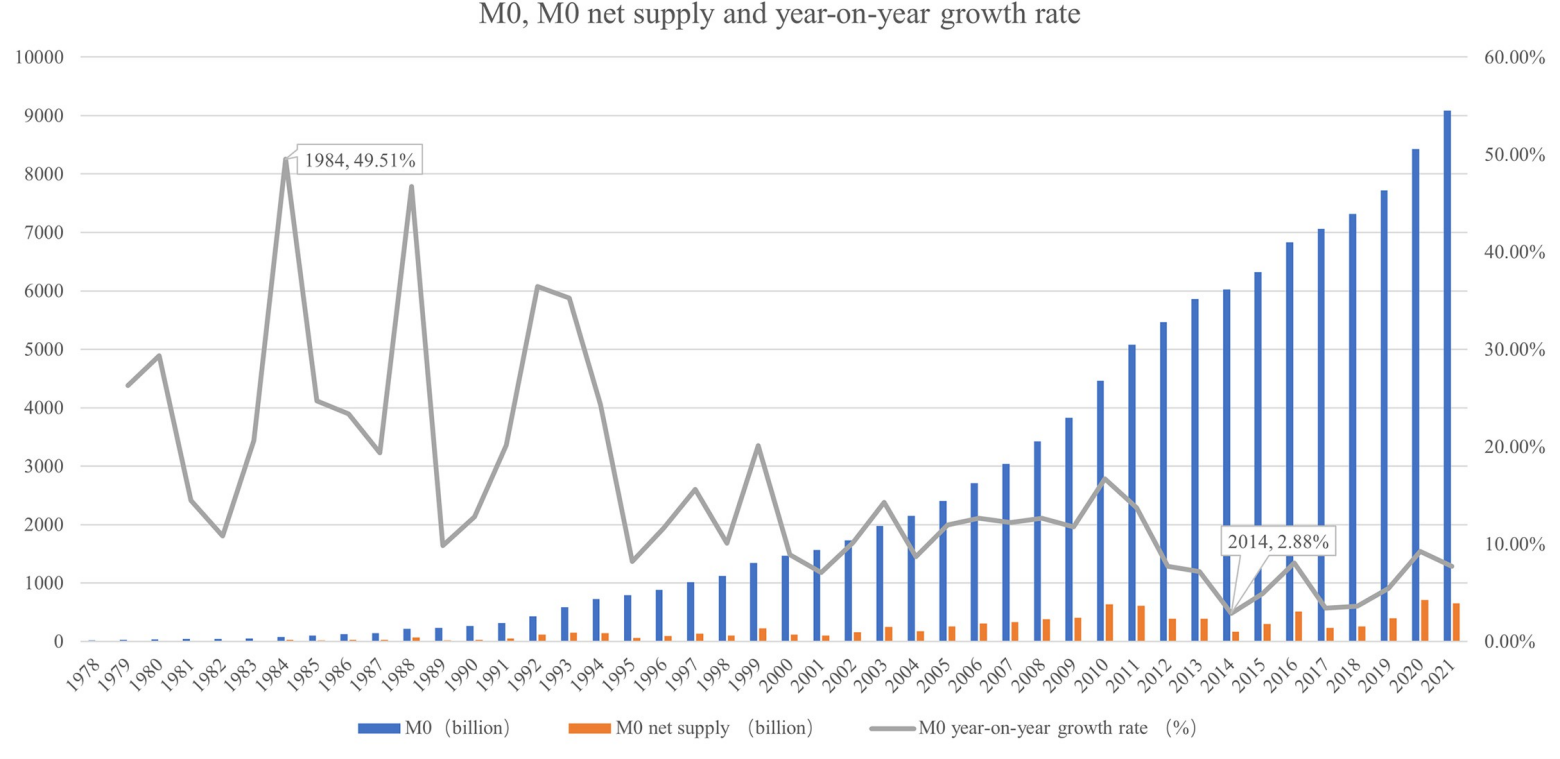
Net supply and year on year growth of M0 and M0. Source: People’s Bank of China.

The changing trend of cash M0 in Circulation in China is shown in Fig 2: Since the reform and opening up in 1978, with the rapid development of the economy, the total amount of M0 has continued to increase from 21.2 billion yuan in 1978 to 9.08 trillion yuan in 2021, with a cumulative increase of 428 times in 43 years and an average annual growth of 15.13%. Since 2012, the growth rate of M0 has decreased significantly, of which the growth rate in 2014 was the lowest, 2.88%, and the growth rate has rebounded slightly in recent years. Although the absolute number of M0 is still increasing, the net supply and growth rate show an obvious downward trend. This change reflects the increasingly obvious substitution effect of the rapid development of debit and credit cards and third-party payments on traditional cash.

E-CNY has the advantages of solid credit endorsement, huge user base, stable user habits and well-established theoretical framework. Firstly, compared with the high circulation cost of traditional CNY, it is more convenient to use, and has no handling fee, just like the traditional CNY. Secondly, unlike traditional third-party payment, which only supports single offline payment, it has the advantages of "dual offline payment", that is, no network is required, and both the payer and the payer can pay offline. Thirdly, it follows the principle of managed anonymity–"anonymity for small value and traceable for high value " [18], and attaches great importance to protecting personal information and privacy. The e-CNY system collects less transaction information than traditional electronic payment and does not provide information to third parties or other government agencies unless stipulated otherwise in laws and regulations. Fourthly, it can be used only by installing the "e-CNY app", and does not need to be "tightly coupled" with bank accounts. Therefore, it can make up for the shortcomings of the existing payment system, better meet diversified payment needs, and will replace most traditional CNY in the future.

**Fig 3:**
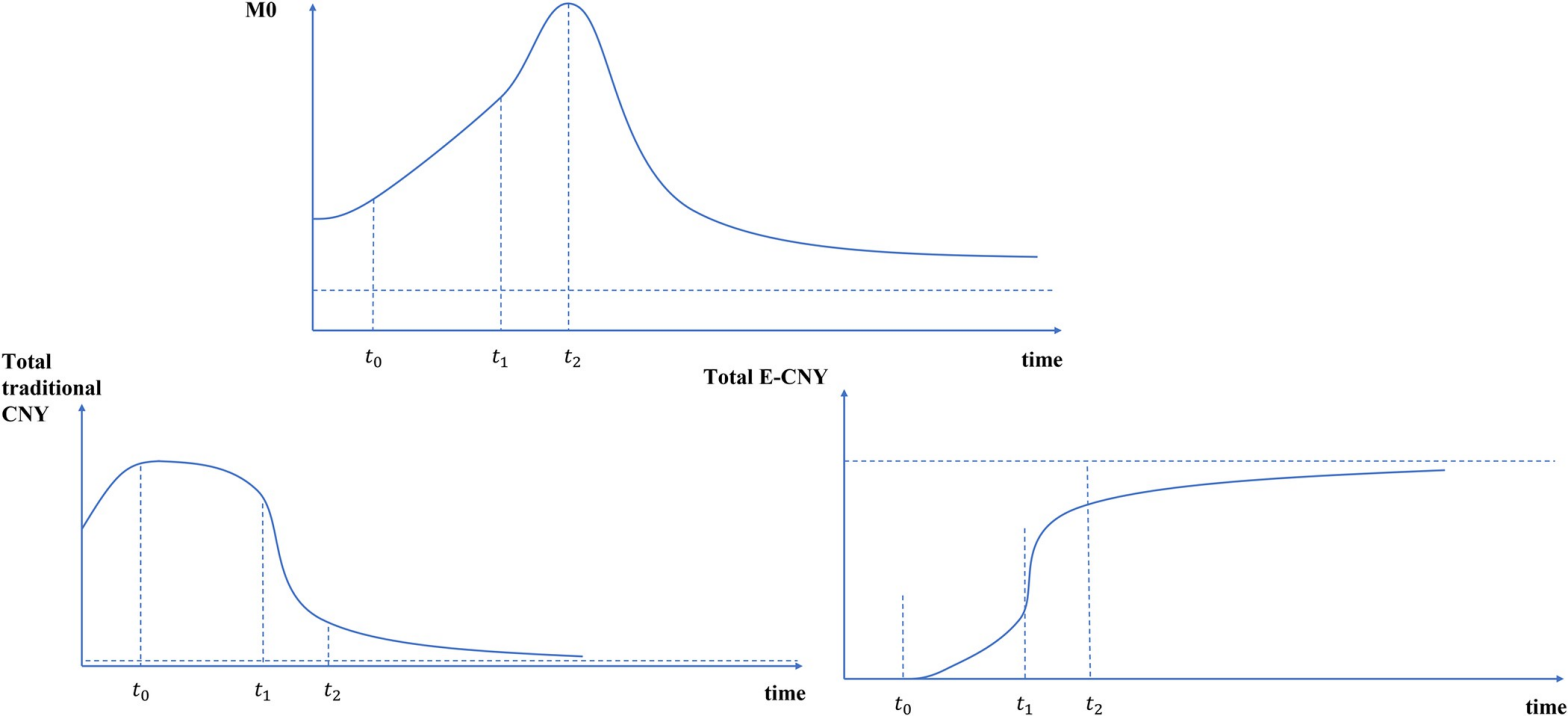
Path changes of M0 after e-CNY issuance.

In the e-CNY era, the total amount of traditional CNY, e-CNY and currency in circulation (M0) which including both will show the change path as shown in Fig 3.

At the beginning of the public pilot of e-CNY, the participation enthusiasm of businesses and consumers will be high. It can be predicted that with the official issuance of e-CNY (*t*_0_), at first, the public will convert some cash into e-CNY (through channel 1) for trading motivation to do some experimental transactions. At this time, e-CNY will slowly replace traditional CNY. With the continuous promotion of e-CNY, the continuous expansion of use scenarios and the continuous improvement of user stickiness, the public’s awareness of the advantages and convenience of e-CNY will become stronger and stronger. At this time (*t*_1_), the transactional demand and preventive demand for traditional CNY will decline, and a growing people will choose to convert traditional CNY into e-CNY, therefore, the substitution effect of e-CNY will be more significant. When e-CNY is widely popularized, people form a consensus to use e-CNY (*t*_2_), and the substitution effect will gradually slow down. Because the liquidity of e-CNY is higher than that of other payment instruments, it is easier to convert into savings, investment and consumption. The transactional demand for e-CNY will gradually stabilize, and the total amount of e-CNY will tend to be stable. In order to meet the needs of the elderly and other groups that do not adapt to intelligent means, traditional CNY will coexist with e-CNY for a long time and maintain a low issuance level. In general, the demand for cash in circulation will steadily decline in the future, and the total amount of M0 in the new steady state will be lower than the original level, reaching a new equilibrium.

### 3.2 The structure of money demand will change, with more frequent conversion between M0, M1 and M2

**Fig 4:**
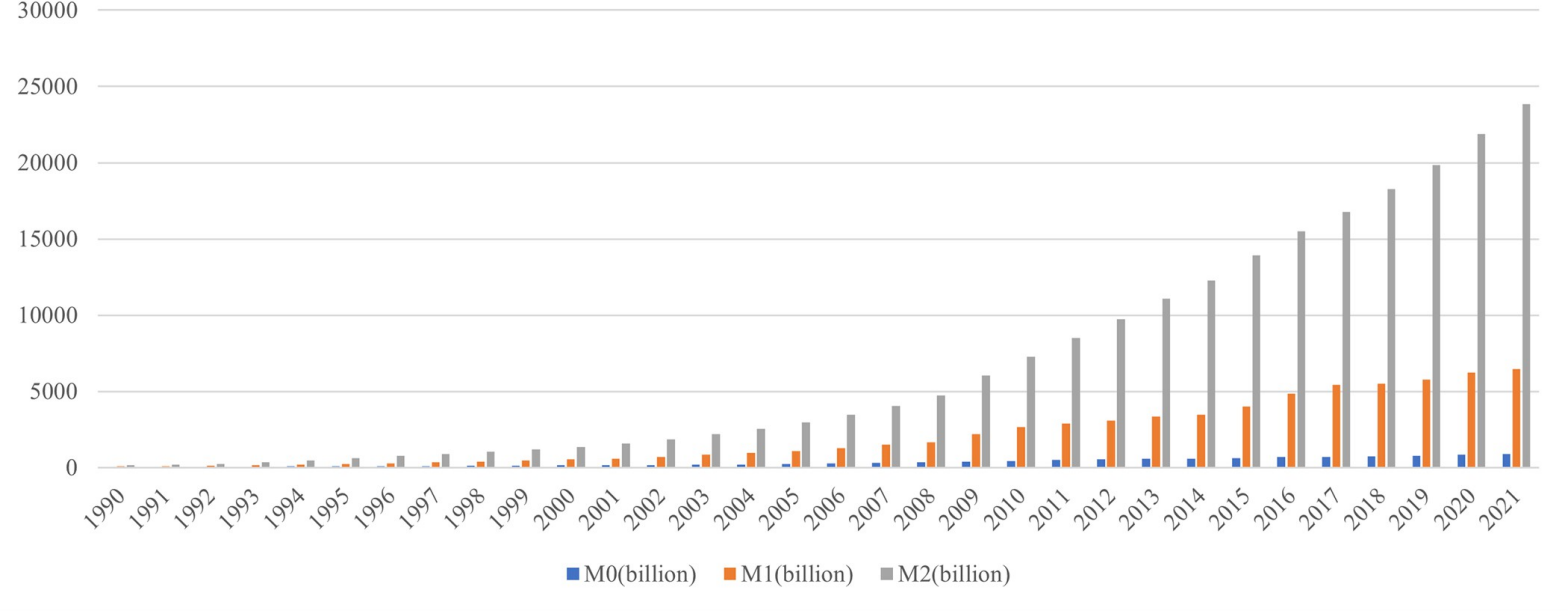
Total supply of M0, M1 and M2. Source: People’s Bank of China.

At present, M1 and M2 in China present a trend of rapid growth, as shown in Fig 4. The growth of M1 and M2 from 695.07 billion yuan and 1529.34 billion yuan in 1990 to 64.74 trillion yuan and 238.297 trillion yuan in 2021 respectively, and is 93 times and 156 times in 31 years, with an average annual growth rate of 15.75% and 17.69% respectively, both faster than the growth rate of M0.

**Fig 5:**
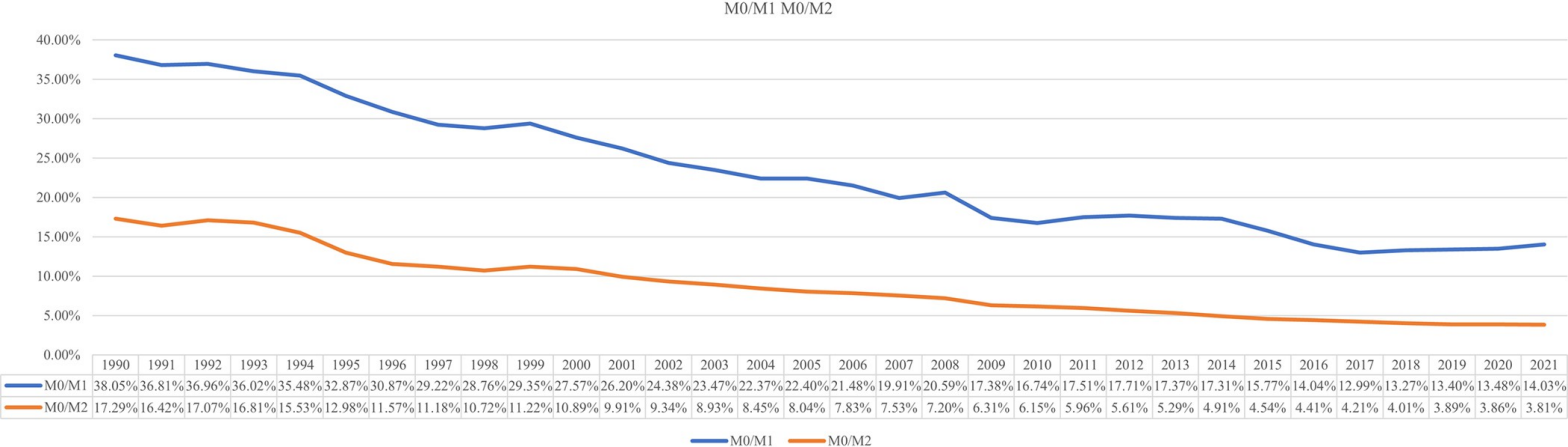
M0/M1 M0/M2 changing trend. Source: People’s Bank of China.

From 1990 to 2021, the proportion of monetary aggregates kept declining, as shown in Fig 5, M0/M1 decreases from 38.05% to 14.03%. M0/M2 decreased from 17.29% to 3.81%, both showed a decrease of more than 70%, and kept falling. This is mainly because the rapid development of non-cash payment instruments had a strong substitution effect on cash.

With the issuance of e-CNY, the demand structure of money will change.

#### 3.2.1 E-CNY may have a certain squeeze on demand deposits

**Fig 6:**
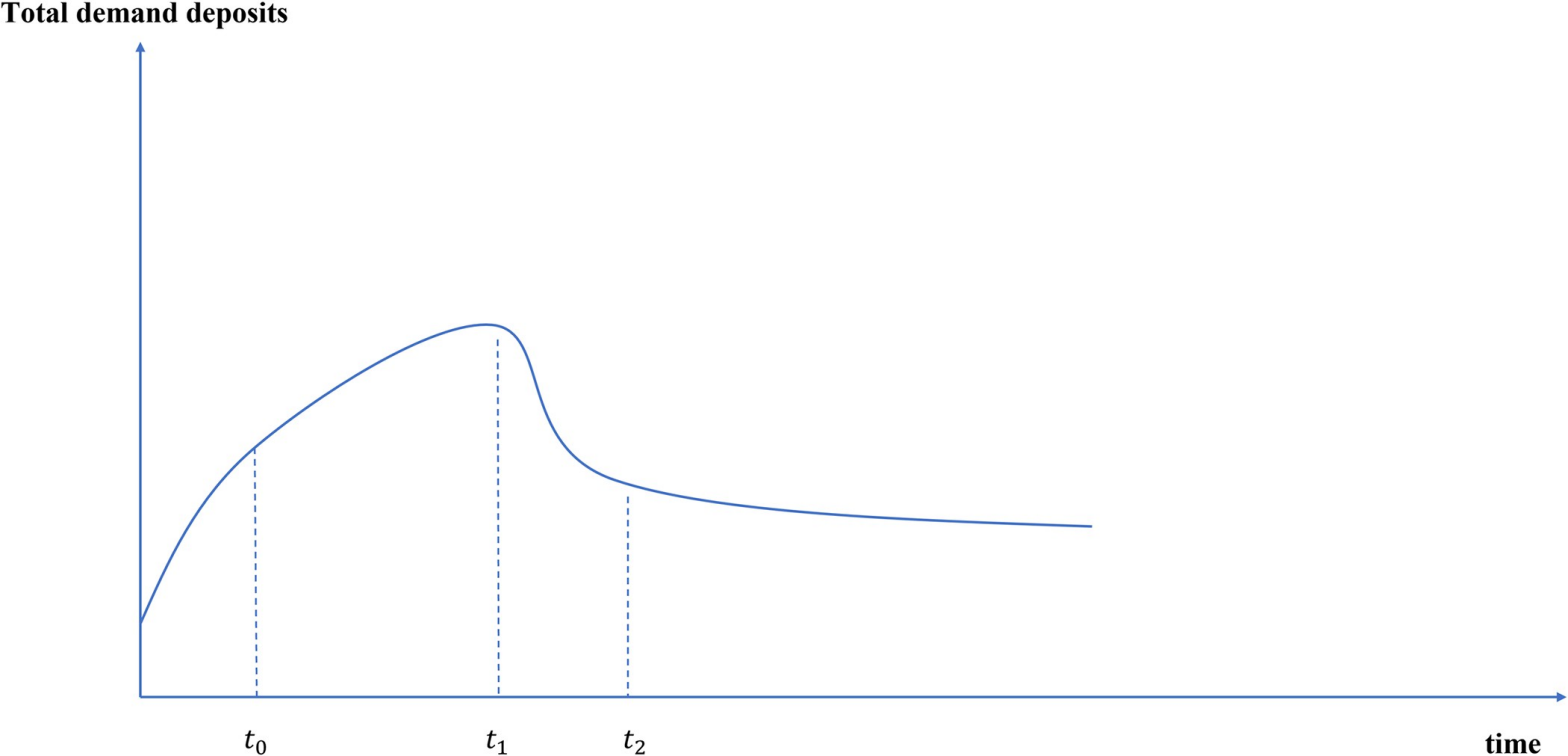
Path changes of demand deposit path after e-CNY issuance.

People usually hold a certain demand deposit to meet the needs of daily transactions. The impact of the issuance of e-CNY on it is shown in Fig 6. At the beginning of the issuance of e-CNY (*t*_0_), because no interest is paid, its substitution effect on demand deposits will not be significant. Demand deposits will grow steadily according to the current trend, but the growth rate will slow down. However, when people’s beneficial perception of the security, convenience and efficiency brought by e-CNY exceeds the meager interest of demand deposits (*t*_1_), people will be more willing to take the initiative to convert some demand deposits into e-CNY (through channel 2), which will squeeze demand deposits to a certain extent. In addition, there will be more daily scenarios (through channel 3) using e-CNY payment such as wages and red envelope subsidies in the future (*t*_2_), although there is no transaction fee in e-CNY wallet, functions such as withdrawal and transfer between accounts will bring time costs to users. In order to reduce the time cost of conversion, people will also be willing to hold a certain amount of e-CNY to meet their daily needs.

#### 3.2.2 E-CNY will have no obvious substitution effect on time deposits and may have an impact on money market funds

At present, M2 in China is mainly composed of M0, enterprise deposits, resident deposits, deposits of non-financial sectors (such as security company users’ margin deposits, insurance deposits, etc.) and money market funds held by non-depositing institutions. The issuance of non-interest-paying e-CNY will have little impact on time deposits with higher interest rates, since the holders of time deposits must give up their demand for liquidity when they choose to get higher interest returns. The design and issuance of e-CNY is to better meet the needs of liquidity, so it will not squeeze time deposits too much. Due to the high liquidity and electronic characteristics of e-CNY, it has a stronger ability to convert into other financial instruments than traditional CNY. Therefore, it may promote the increase of deposits in the non-financial sector. In terms of the money market funds such as Yu’e Bao, the yield has decreased significantly from the beginning, from the highest annualized rate of more than 7% to the current rate of about 2%. Although these money market funds can meet the liquidity and profitability requirements of users, when encountering large market fluctuations, some users may tend to hold risk-free e-CNY for hedging out of safety considerations. In this case, some M2 will be converted to M0.

### 3.3 The velocity of money will be faster and the circulation efficiency will be improved

**Fig 7:**
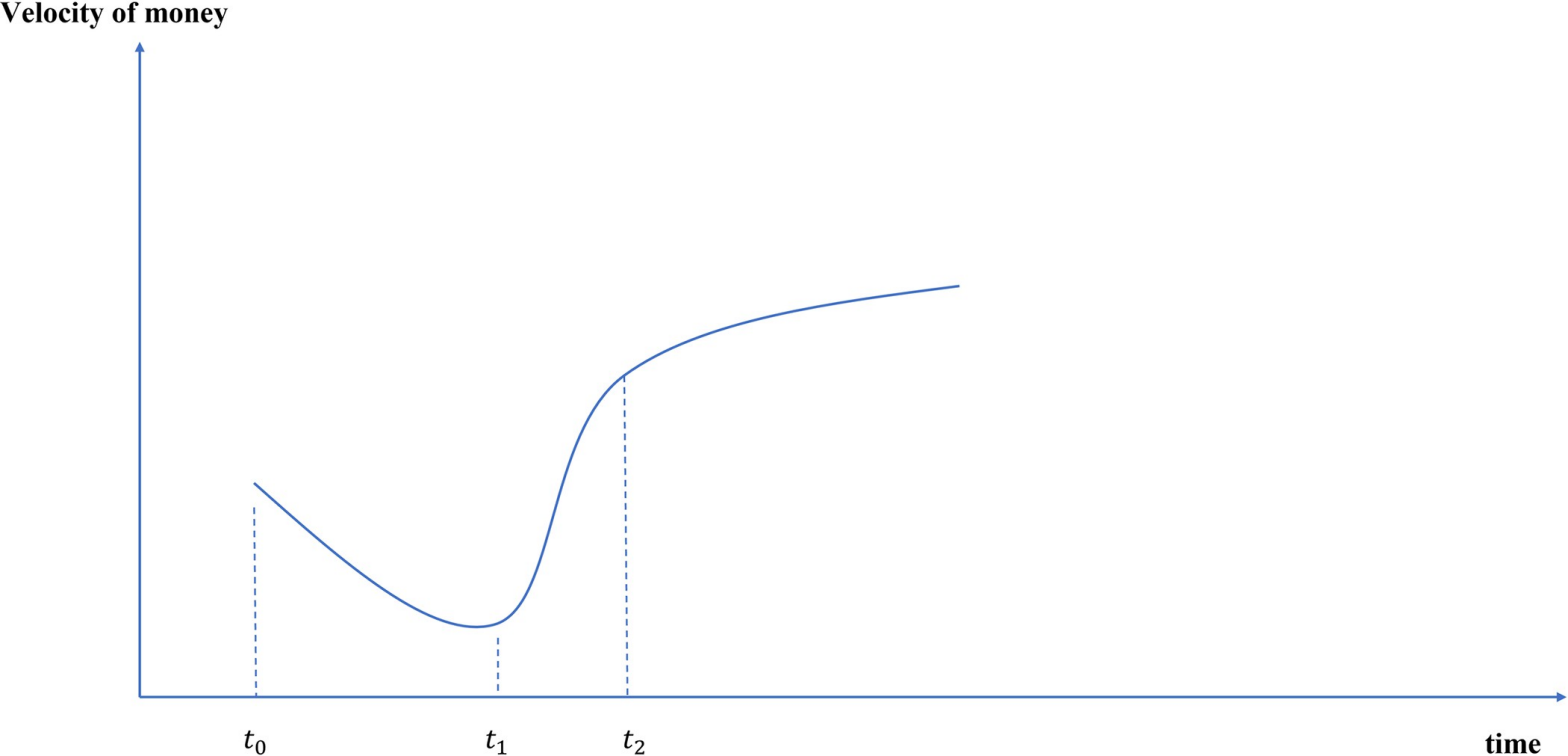
Path changes of currency velocity after e-CNY issuance.

According to Fisher’s trading equation, *MV* = *PY*, after issuing e-CNY, the velocity of money will follow:

$$M^{c}V^{c}+M^{e}V^{e}=PY$$ (1)

where *M*^*c*^ is the quantity of traditional CNY, *V*^*c*^ its velocity, *M*^*e*^ the quantity of e-CNY and *V*^*e*^ the velocity of e-CNY. As mentioned above, the substitution effect of e-CNY on traditional CNY, *M*^*c*^ will decrease, and accordingly, *V*^*c*^ will also tend to decrease. In the early stage of e-CNY (*t*_0_), as the traditional CNY still accounts for a large proportion, *V*^*c*^ will be dominated and show a downward trend. With the increasing popularity of e-CNY (*t*_1_), *M*^*e*^ will increase. Due to the stronger liquidity of e-CNY, it is easier to convert to other currency forms, and its velocity *V*^*e*^ is faster than that of traditional CNY *V*^*c*^. With the wide application of e-CNY, the overall velocity will show an upward trend as *V*^*e*^. Therefore, the overall currency velocity will show a V-shaped trend with the development of e-CNY, and eventually tend to be stable, as shown in Fig 7.

Given that e-CNY and e-money have many common characteristics, several empirical evidence on electronic money shows that the wide use e-money will speed up velocity [65–67]. When the digital renminbi is issued on a large scale and the released data is more detailed, more rigorous empirical evidence is needed to examine its impact on the currency velocity.

## 4. The impact of e-CNY on money supply

With the issuance of e-CNY, the money supply and money multiplier may change, and China’s money supply will change in many aspects.

### 4.1 The impact of e-CNY on the total amount of money

#### 4.1.1 The reserve of the central bank will be more controllable, the deposit reserve will reduce, and the total reserve will increase

The central bank implements the deposit reserve system to ensure the liquidity of commercial banks and other depository monetary institutions, and prevent the risk of run. At present, China’s deposit reserve is made up of reserve requirements and excess deposit reserve. In the future, e-CNY reserve will also become the reserve that commercial banks deposit at the central bank.

First, as mentioned above, e-CNY will replace most of the traditional CNY. E-CNY requires equal exchange of traditional CNY in commercial bank accounts, that can be regarded as 100% full payment of e-CNY reserve, which will increase the reserve of the central bank. Based on the intelligent and digital characteristics of e-CNY issuance, the reserve of this part is controllable since the number of e-CNY to be issued is monitored by the central bank. Secondly, as e-CNY will crowd out some demand deposits, the amount of demand deposit reserve of commercial banks will decrease accordingly. Since the e-CNY reserve is paid in full, and its reserve ratio is higher than the reserve requirements ratio of demand deposits, the decline of demand deposit reserve will be much lower than that of e-CNY reserve. Third, with regard to excess reserves, after the issuance of e-CNY, the ability of commercial banks to control liquidity risk will also be improved due to the application of big data and other technologies, which will enhance the ability to allocate monetary resources, and the number of excess reserves will be reduced to a certain extent.

**Fig 8:**
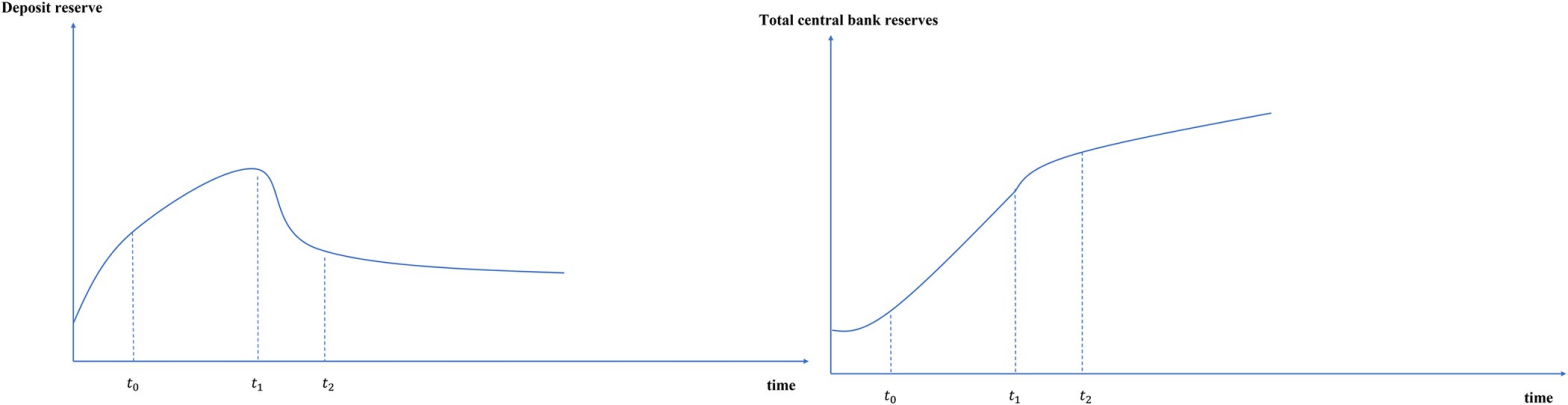
Path changes of the central bank reserve after e-CNY issuance.

Based on the above three aspects, the issuance of e-CNY will generally promote the number of reserves, and the path change is shown in Fig 8. With the official issuance of e-CNY (*t*_0_), the reserve of e-CNY will continue to increase, and with the partial extrusion of e-CNY to demand deposits (*t*_1_), the reserve of demand deposits will decrease, but the decline rate is lower than the growth rate of e-CNY reserve. When the e-CNY is widely popularized (*t*_2_), the growth rate of reserves will gradually slow down and become stable.

#### 4.1.2 The monetary base and the demand for cash in treasury will decrease

The Monetary base consists of currency held by resident departments, reserve requirements, excess deposit reserve and cash in treasury of commercial banks. Based on the above analysis, as the issuance and withdrawal of e-CNY will be more intelligent and better adapted to economic conditions, the amount of monetary base will decrease compared with that before the issuance of e-CNY. Due to the substitution effect and crowding out effect, the reserve requirements and excess deposit reserve will also decrease accordingly. As for cash in treasury, the issuance of e-CNY makes the management efficiency of currency demand constantly improve, and the demand for cash in treasury will decrease.

**Fig 9:**
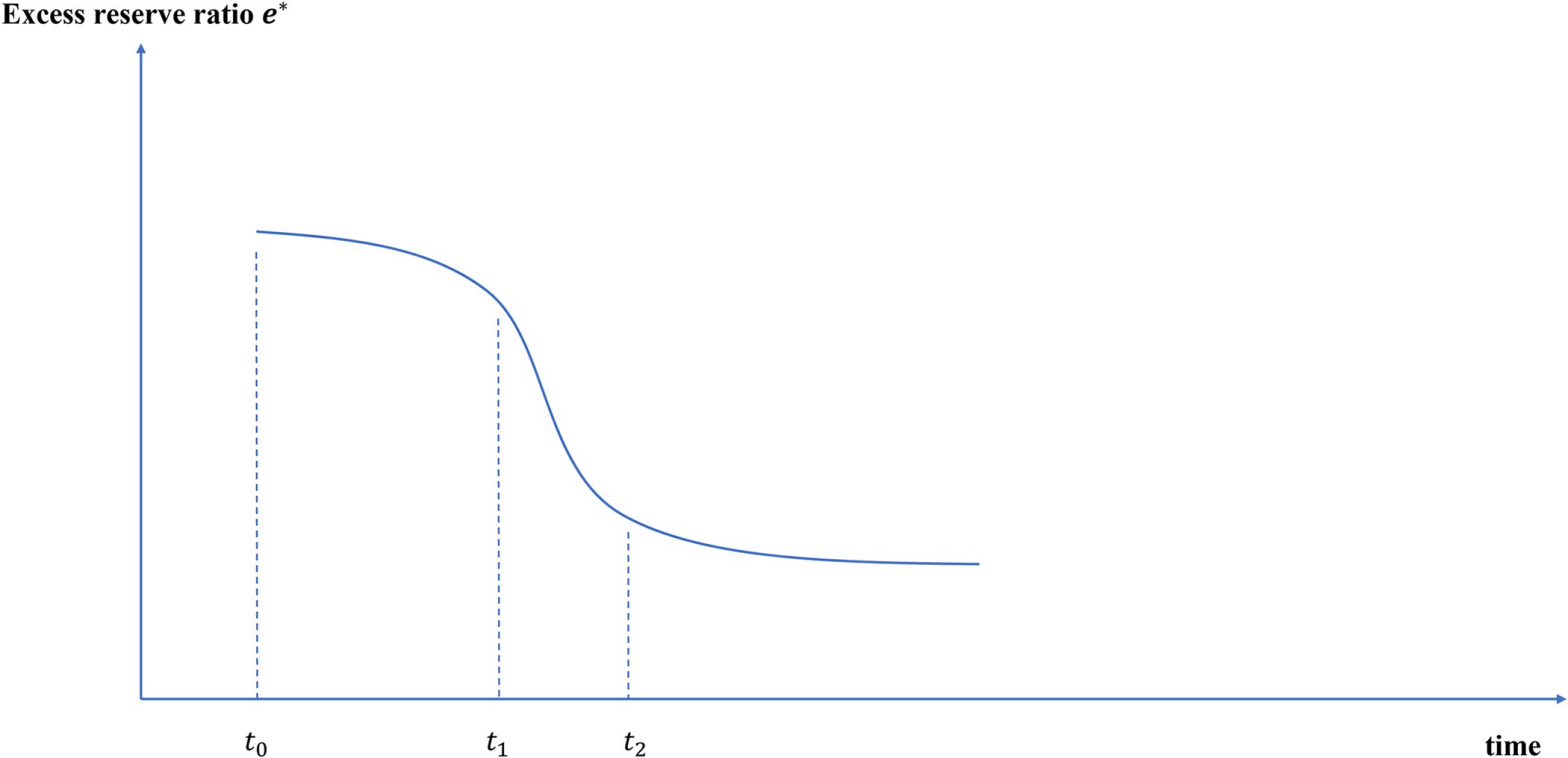
Path changes of monetary base after e-CNY issuance.

Therefore, the amount of monetary base will steadily decline, as shown in Fig 9. With the official issuance of e-CNY (*t*_0_), the quantity of monetary base will decline slowly. As e-CNY substitutes for traditional CNY and demand deposit more significantly (*t*_1_), the decline rate of monetary base will increase. When e-CNY is widely popularized (*t*_2_), it will gradually slow down and become stable.

### 4.2 The influence of e-CNY on currency multiplier

#### 4.2.1 The money multiplier expression will change

As mentioned in the second part, the issuance of e-CNY may have an impact on the total amount of money supply and base money, and the money multiplier will also be affected by the issuance of e-CNY.

In the era of traditional CNY, the base currency can be expressed as:

$$B={C+RR+ER=C}_{c}+RR+ER+C_{B}$$ (2)

Among them *C* is currency in circulation, which is composed of currency held by the public *C*_*c*_ and cash in treasury held by commercial banks *C*_*B*_. *RR* is reserve requirements and *ER* excess reserve. Specifically:

$$B=C+RR+ER=C+D\times r_{d}+T\times r_{d}+E\times r_{e}$$ (3)

Among them, *D*, *T*, *E* are the scale of demand deposits, time deposits and excess reserves respectively, *r*_*d*_, *r*_*e*_ are reserve requirement ratio and excess reserve ratio respectively, and *r*_*e*_<*r*_*d*_.

Assuming the cash leakage rate $k=\frac{C}{D}$, the ratio of time deposit to demand deposit is $t=\frac{T}{D}$, and the ratio of excess reserve to demand deposit is $e=\frac{E}{D}$.

In this case, the narrow money multiplier is:

$$m_{1}=\frac{C+D}{C+RR+ER}=\frac{1+k}{k+{(1+t)r}_{d}+er_{e}}$$ (4)

The broad money multiplier is:

$$m_{2}=\frac{C+D+T}{C+RR+ER}=\frac{1+k+t}{k+{(1+t)r}_{d}+er_{e}}$$ (5)

After the issuance of e-CNY, it will replace part of the traditional CNY in the hands of the public, squeeze deposits to a certain extent, reduce the scale of excess reserves and reduce the cash in treasury of commercial banks.

Assume that the amount of e-CNY is *P*, the total amount of cash currency issued on a large scale by e-CNY is reduced from *C* to C*, demand deposit is reduced from *D* to D*, part of time deposit is reduced from *T* to T*, and excess reserve is reduced from *E* to E*.

Assume that after the issuance of e-CNY, the cash leakage rate is $k^{*}=\frac{C^{*}+P}{D^{*}}$, the ratio of the sum of traditional CNY and e-CNY to deposits, the ratio of time deposit to demand deposit is $t^{*}=\frac{T^{*}}{D^{*}}$, and the ratio of excess reserve to demand deposit is $e^{*}=\frac{E^{*}}{D^{*}}$.

Then, after the issuance of e-CNY, the new multiplier of narrow and broad money is:

$${m_{1}}^{*}=\frac{1+k^{*}}{k^{*}+{(1+t^{*})r}_{d}+e^{*}r_{e}}$$ (6)

$${m_{2}}^{*}=\frac{1+k^{*}+t^{*}}{k^{*}+{(1+t^{*})r}_{d}+e^{*}r_{e}}$$ (7)

#### 4.2.2 Cash leakage k* will increase first and then decrease, increasing money multiplier fluctuations and expansionary effects

Take the derivative of the cash leakage rate k* in Eq (6) and Eq (7), and get Eq (8) and Eq (9):

$$\frac{\partial {m_{1}}^{*}}{\partial k^{*}}=\frac{{(1+t^{*})r}_{d}+e^{*}r_{e}-1}{{\left[k^{*}+{(1+t^{*})r}_{d}+e^{*}r_{e}\right]}^{2}}$$ (8)

$$\frac{\partial {m_{2}}^{*}}{\partial k^{*}}=\frac{{(1+t^{*})(r}_{d}-1)+e^{*}r_{e}}{{\left[k^{*}+{(1+t^{*})r}_{d}+e^{*}r_{e}\right]}^{2}}<0$$ (9)

In Eq (8), referring to the current weighted average deposit reserve ratio of financial institutions is 8.9% and excess reserve ratio is 0.35%, according to the latest data of the People’s Bank of China, the ratio of time deposits to demand deposits in the first half of 2020 is about 5 (usually kept between 2–5), substituted into E (8), $\frac{\partial {m_{1}}^{*}}{\partial k^{*}}<0$; Cash leakage rate is negatively correlated with narrow money multiplier. In Eq (9), since r_d_<1, $\frac{\partial {m_{2}}^{*}}{\partial k^{*}}<0$, the cash leakage rate is negatively correlated with the broad money multiplier.

**Fig 10:**
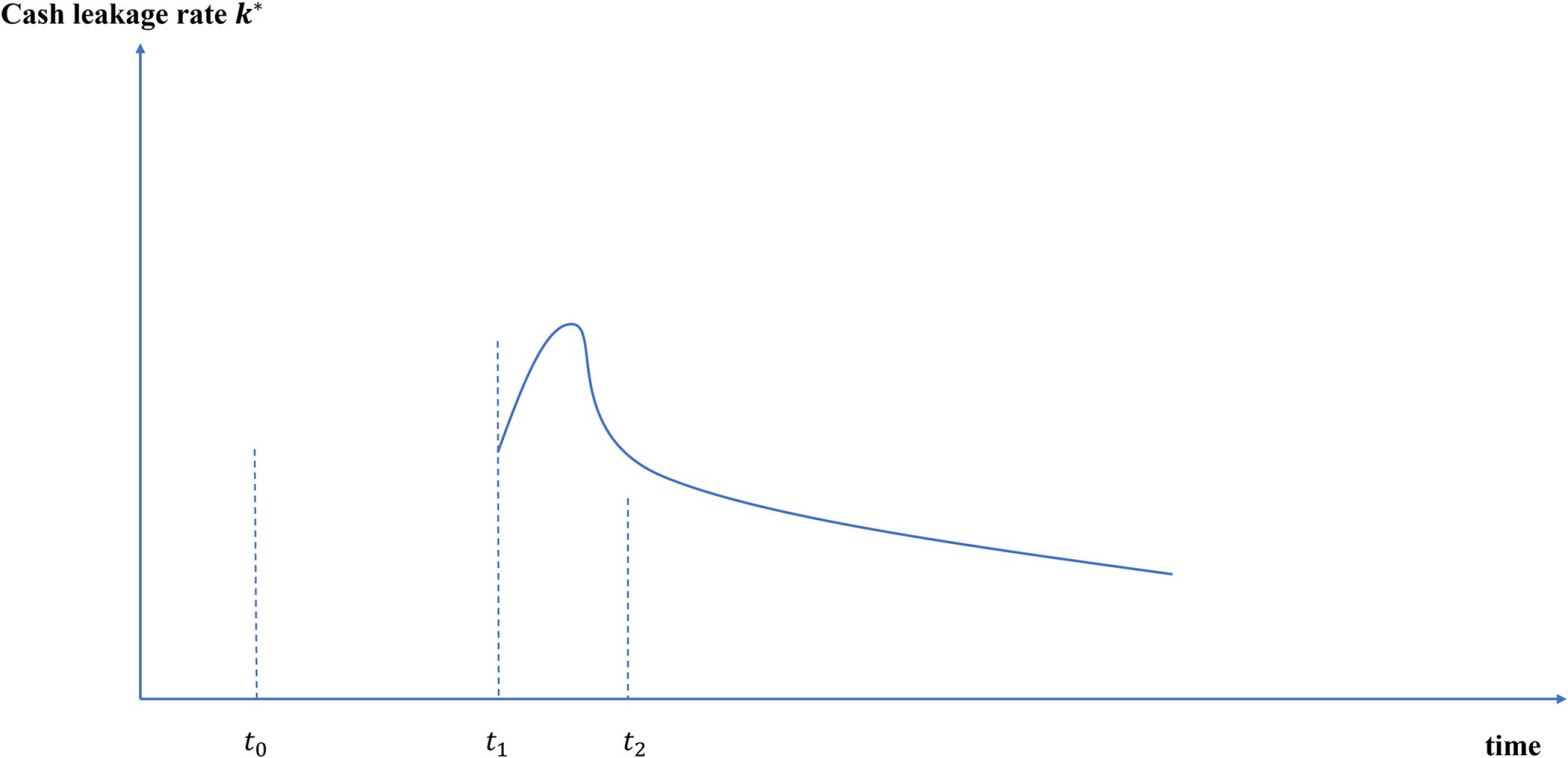
Path changes of cash leakage rate after e-CNY issuance.

Investigate the change path of cash leakage rate k* after issuing e-CNY. At the beginning of the issuance of e-CNY (*t*_0_), the substitution effect of e-CNY on demand deposits will not be significant. As e-CNY becomes more recognized and used (*t*_1_), people will convert part of their demand deposits into e-CNY, which k* will increase. In the future, when e-CNY can fully meet people’s liquidity needs (*t*_2_), k* will gradually stabilize and be generally lower than before the issuance of e-CNY, as shown in Fig 10.

Due to the negative correlation between the cash leakage rate and the narrow broad money multiplier, the issuance of e-CNY will have a contraction effect on the money multiplier in the short run, and will make the money multiplier fluctuate and show an expanding trend in the long run.

#### 4.2.3 The ratio of time deposits t* will gradually increase, the narrow money multiplier will contract, and the broad money multiplier will expand

Derivative of the time deposit ratio t* in Eqs (6), (7), (10) and (11) is obtained:

$$\frac{\partial {m_{1}}^{*}}{\partial t^{*}}=\frac{{-(1+k^{*})r}_{d}}{{\left[k^{*}+{(1+t^{*})r}_{d}+e^{*}r_{e}\right]}^{2}}<0$$ (10)

$$\frac{\partial {m_{2}}^{*}}{\partial t^{*}}=\frac{{k^{*}(1-r}_{d})+e^{*}r_{e}}{{\left[k^{*}+{(1+t^{*})r}_{d}+e^{*}r_{e}\right]}^{2}}>0$$ (11)

According to Eqs (10) and (11), time deposit ratio has a negative effect on the narrow money multiplier and a positive effect on the broad money multiplier.

**Fig 11:**
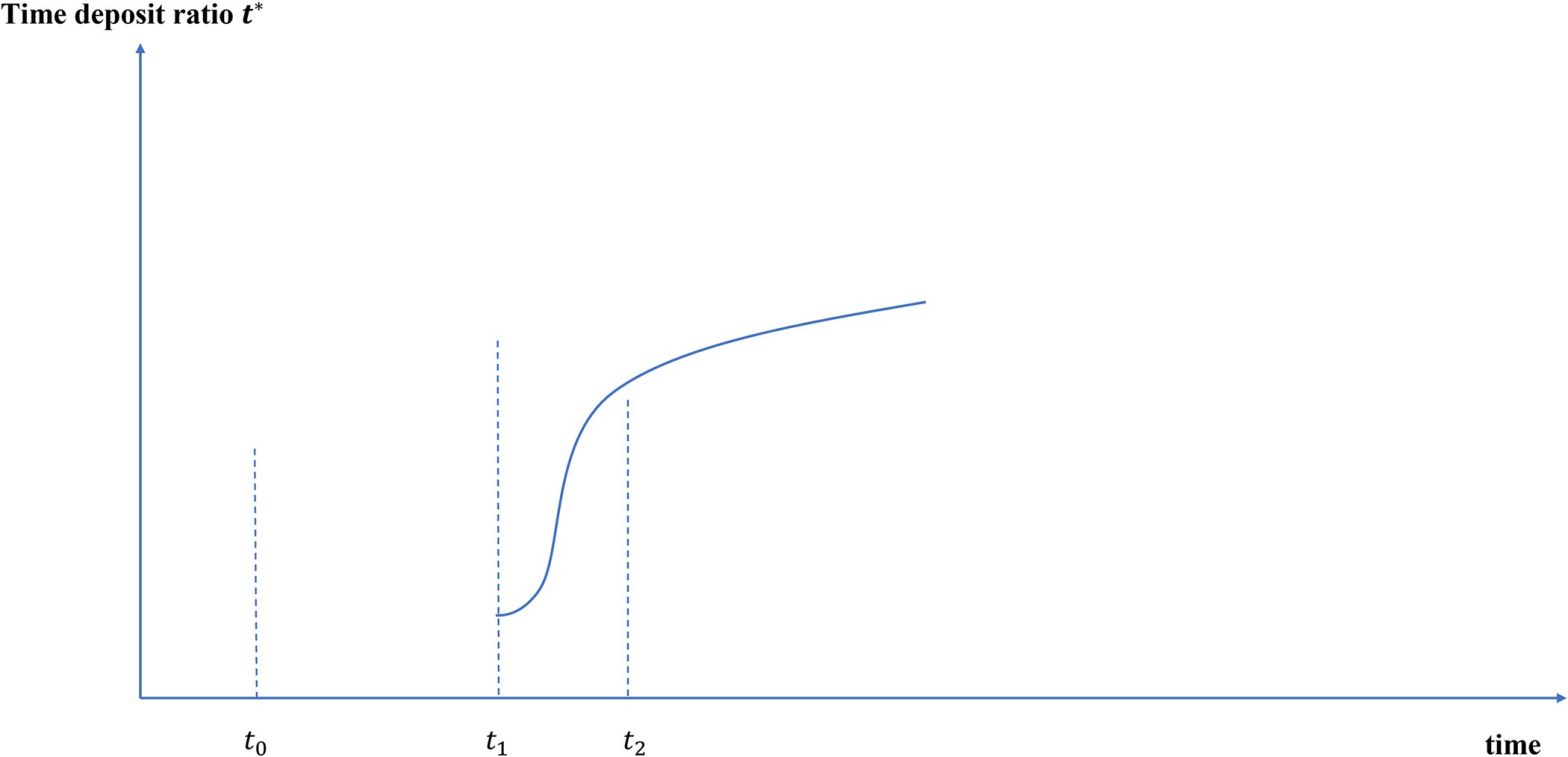
Path changes of time deposit ratio after e-CNY issuance.

As mentioned in the second part, the impact of non-interest-bearing e-CNY on time deposits with high interest income is limited. After the acceptance of e-CNY becomes higher (*t*_1_), there will be a certain squeeze on demand deposits with low interest income. Therefore, the time deposit ratio t* will not change much in the short term and will rise in the medium and long term, as shown in Fig 11.

According to Eqs (10) and (11), the issuance of e-CNY will shrink the narrow money multiplier and expand the broad money multiplier in the medium and long term.

#### 4.2.4 The excess reserve ratio e* will gradually decrease and the money multiplier will gradually expand

The excess reserve ratio e* in Eqs (6) and (7) is derived to obtain Eqs (12) and (13):

$$\frac{\partial {m_{1}}^{*}}{\partial e^{*}}=\frac{{-(1+k^{*})r}_{e}}{{\left[k^{*}+{(1+t^{*})r}_{d}+e^{*}r_{e}\right]}^{2}}<0$$ (12)

$$\frac{\partial {m_{2}}^{*}}{\partial e^{*}}=\frac{-(1+k^{*}+t^{*})r_{e}}{{\left[k^{*}+{(1+t^{*})r}_{d}+e^{*}r_{e}\right]}^{2}}<0$$ (13)

According to Eqs (12) and (13), the excess reserve ratio has a negative effect on both narrow and broad money multipliers.

**Fig 12:**
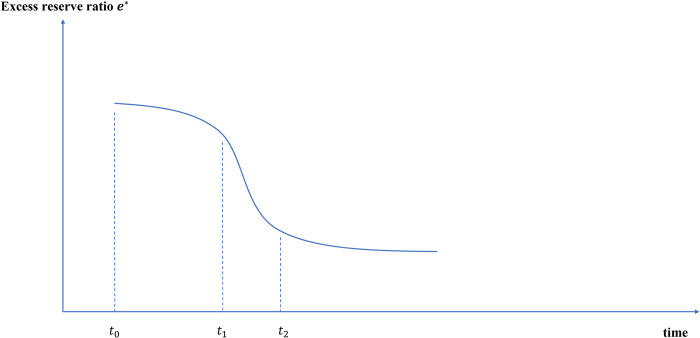
Path changes of excess reserve ratio after e-CNY issuance.

After the issuance of e-CNY (*t*_0_), as the money supply is more intelligent, the fund management efficiency of commercial banks will be further improved, the number of excess reserves will be reduced to a certain extent, and the excess reserve ratio will be reduced, as shown in Fig 12.

According to Eqs (12) and (13), after the issuance of e-CNY, the money multiplier will continue to rise and eventually tend to be stable.

To sum up, the issuance of e-CNY may have a complex and multifaceted impact on the money multiplier. Generally speaking, it will increase the volatility of the narrow and broad money multiplier, and will have an overall expansionary effect on the money multiplier. This is consistent with the conclusions of existing studies [68–70].

## 5. The influence of e-CNY on monetary policy transmission mechanism

The People’s Bank of China has applied for many patents for legal digital currency, among which four forward-looking condition triggering mechanisms (shown in Table 1) are set up, which is conducive to the transmission of monetary policy [71–74].

**Table 1:** E-CNY’s forward-looking condition triggering mechanisms.

Triggering Conditions	Setting Propose
**The time-point conditional triggering management method**	Control the issuance time of e-CNY
**The specific entity conditional triggering management method**	Control the flowing entity of e-CNY and avoid possible moral hazard problems in commercial banks
**The loan interest rate conditional triggering management method**	Control the real interest rate at which real enterprises obtain loans and avoid decoupling between the central bank’s benchmark interest rate and market interest rates
**The economic state conditional triggering management method**	Control the interest rate when commercial banks return e-CNY to the central bank, which can smooth economic fluctuations and avoid financial risks that may be caused by drastic changes in economic development.

**Fig 13:**
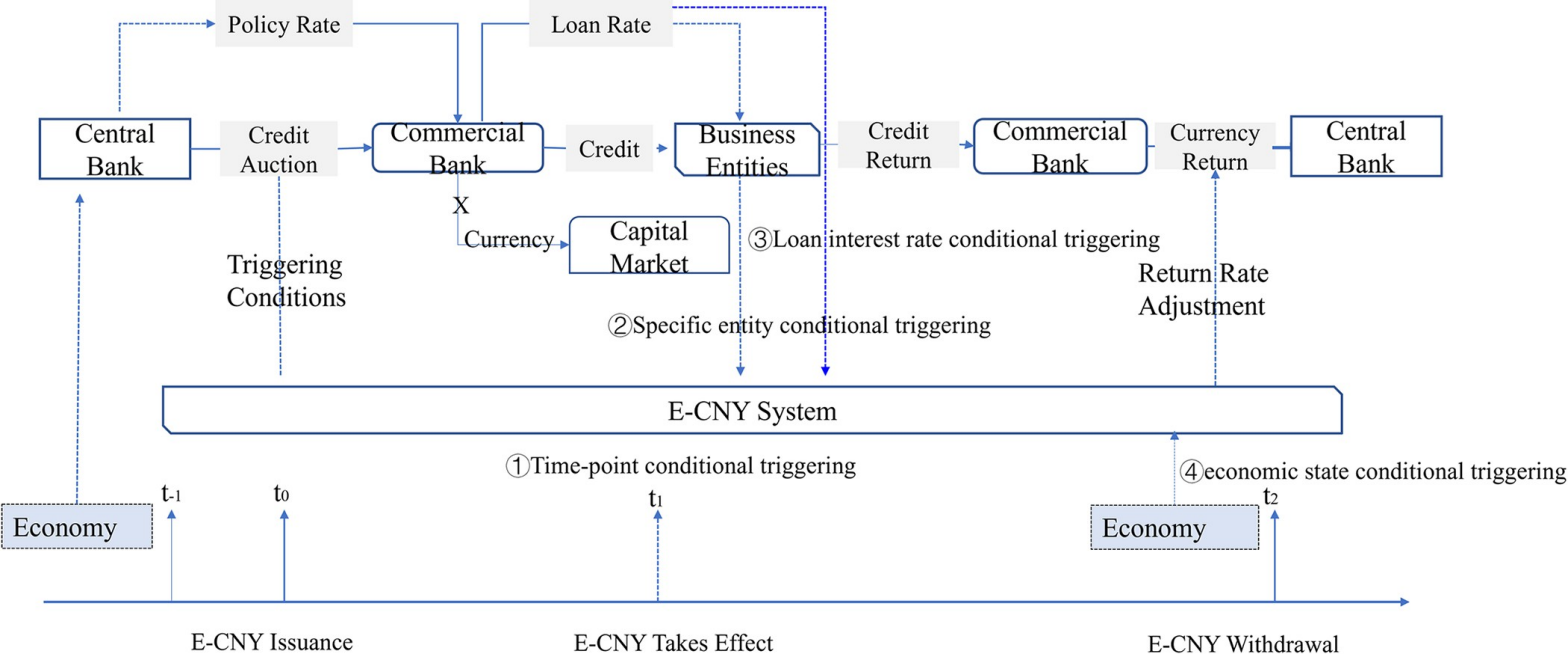
Schematic diagram of the four triggering conditions.

The schematic diagram of the four triggering conditions is shown in the Fig 13.

E-CNY adopts a "forward-looking condition trigger mechanism" when it is issued. Through the four triggering conditions, the effectiveness of the e-CNY can be controlled after its release: if the relevant conditions meet the policy requirements set by the central bank, then the forward-looking conditions can be successfully triggered, and e-CNY will take effect, otherwise it will be just a string of codes and cannot be used.

**Fig 14:**
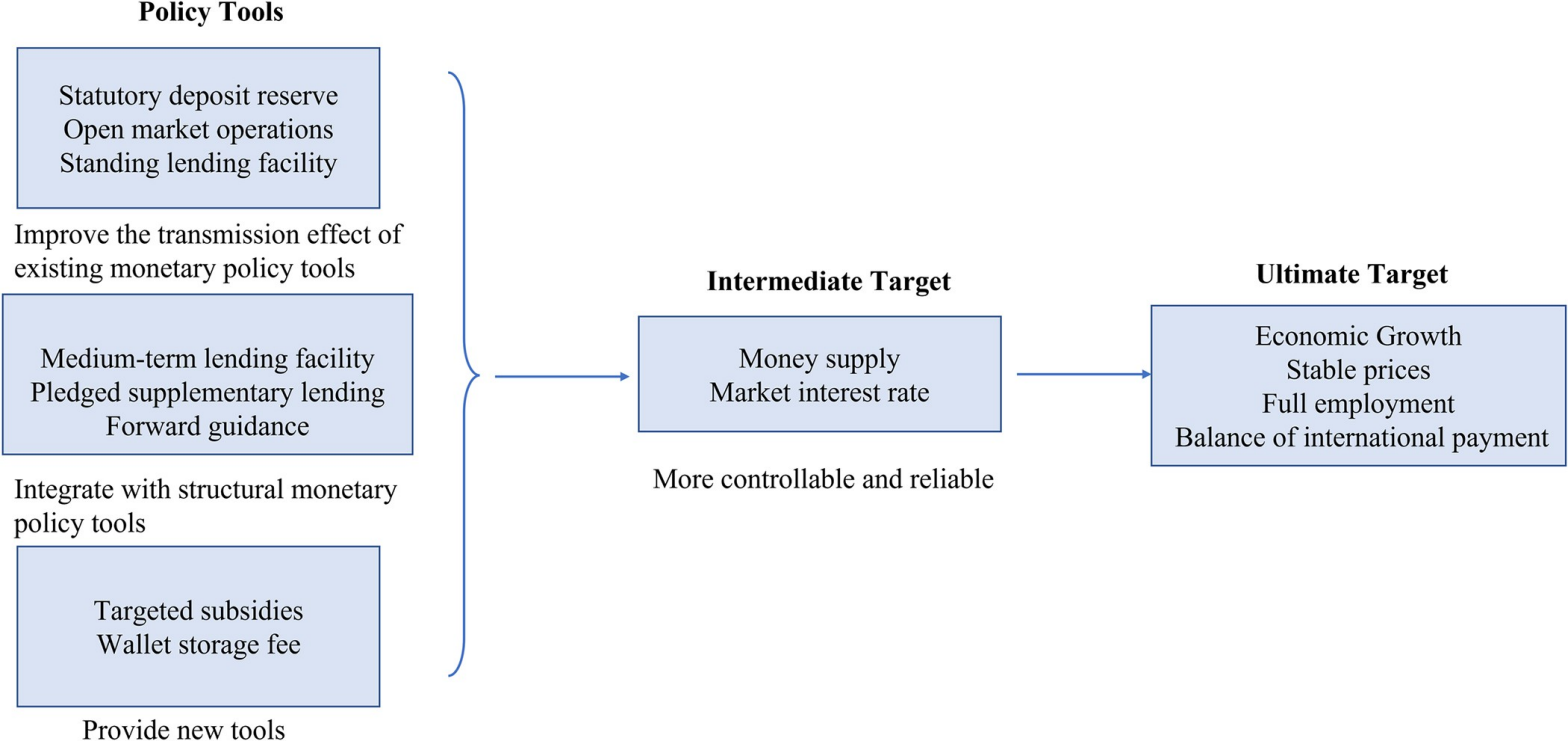
Schematic diagram of the influence of e-CNY on the transmission mechanism of monetary policy.

Due to the special design of the e-CNY, its issuance will improve the transmission mechanism of existing monetary policies from the aspects of monetary policy tools, intermediate targets and ultimate targets, as shown in Fig 14.

### 5.1 The impact of e-CNY on intermediate targets of monetary policy

#### 5.1.1 The impact of the e-CNY on the total money supply

As described above, the issuance of e-CNY may enhance the expansion effect of money multiplier, it may also exert an impact on the supply of base money and increase the volatility of total money supply. Relying on its unique issuing technology, it can better control the amount of e-CNY. In addition, e-CNY has a lot of technical advantages, for example, it can be controllable and managed anonymous, solve the problem of fund payers tracking the flow of funds across entities and layers, track the flow of funds within the management scope of the initiator, protect user privacy, and support customized tracking of currency flow.

#### 5.1.2 Influence of e-CNY on deposited funds in depository account

At present, a large amount of in-transit funds deposit in the depository account during the financing process. Although commercial banks supervise the depository account, it still cannot eliminate the risk of false appropriation of the platform, as well as the operational risk and security risk of the platform. In addition, information asymmetry is serious in this situation. The entire transaction and contract execution process is dominated and controlled by the platform, lacking supervision and guarantee, so there are operational loopholes that the platform may fabricate. The e-CNY may offer a way to invest and fund transactions at the beginning of the design. E-CNY can be used to provide the platform with the payment and settlement of investment and financing fund transfer, realize the direct transfer of money, solve the risk of depository account precipitation fund, reduce the opaque fund path under the depository account mode, and thus solve the operational risk and the main risk of the platform. In addition, the real-time monitoring system can also strengthen the supervision of underground economic activities.

#### 5.1.3 The influence of e-CNY on interest rate transmission lag

The issuance of e-CNY will also shorten the time lag of interest rate channels and better meet the requirements of financial services to the real economy [75]. The application of big data analysis will enhance the ability of the central bank to guide market interest rates through monetary policy tools. It can also help to acquire more and more real-time information, thus making interest rate adjustment more sensitive and more in line with current market conditions. Through the management method based on the conditional trigger of loan interest rate [73], the benchmark interest rate can be effectively transmitted to the loan interest rate in real time, and the problems of monetary policy transmission lag caused by the loss of control over money can be solved. The time-point conditional trigger management method [71] can effectively solve the contemporaneous problem of the current monetary policy operation, so that the time point of currency takes effect is not limited to the current issue of currency, but extended to a certain point in the future in line with the policy objectives, avoiding money idling, and thus reducing the transmission lag of monetary policy.

### 5.2 The influence of e-CNY on the transmission effect of monetary policy tools

#### 5.2.1 Increase the flexibility of open market operations

Due to the intelligent characteristics of e-CNY, its issuance will shorten the time of open market operation, give the central bank real-time feedback ability, and timely control the volume of open market operation [76]. In addition, e-CNY can also support the management method triggered by economic state conditions [74], which can countercyclically adjust the capital return rate of financial institutions to currency issuers according to the economic information at the recovery time point, so as to reduce the procyclicality of risk characteristics and loan behavior of financial institutions, avoid the "liquidity trap" and realize the countercyclical regulation of economy. E-CNY will also improve the flexibility and effectiveness of open market operation to a certain extent, which is more conducive to stabilizing market expectations and effectively preventing financial risks.

#### 5.2.2 Give better play to the advantages of the interest rate corridor of standing lending facility (SLF)

The issuance of e-CNY will increase the velocity and increase the ability of commercial banks to obtain emergency funds. At the same time, it will also enable the central bank to better understand the liquidity needs of financial institutions and provide more targeted "point-to-point" credit support, so as to better play the role of the upper limit of interest rate corridor, smooth out interest rate fluctuations, maintain the smooth operation of money market interest rates and provide appropriate liquidity for the market. At the same time, as e-CNY will improve the transparency of monetary policy and currency liquidity, it is also conducive to the formation of market-oriented interest rates and better construction of interest rate corridor mechanism.

### 5.3 The impact of e-CNY on structural monetary policy tools

On the basis of traditional monetary policy, People’s Bank of China formed a structural monetary policy tool system with Chinese characteristics including two types: quantitative and interest rate oriented. Among them, quantitative structural monetary policy tools pay more attention to structure and directionality, so as to realize the targeted delivery of liquidity, reduce the financing cost of small and micro enterprises, promote the development of "agriculture, rural areas and farmers", and support the development of real economy. The targeted RRR cuts, re-loan to support agriculture, and re-loan to support small and medium-sized enterprises are included in this type. The interest rate-oriented structural monetary policy tool is a lending facilitation tool based on interest rate adjustment, which helps to alleviate the fluctuation of inter-bank interest rates, thereby further improving the transmission mechanism from short-term interest rates to long-term interest rates, stabilizing the overall social financing cost, and guiding the market expectations. Standing Lending Facility (SLF), Medium Term Lending Facility (MLF) and Pledged Supplementary Loan (PSL) are included in this type. The combination of some characteristics of e-CNY and structural monetary policy will make its transmission more efficient.

#### 5.3.1 Targeted money supply, better implementation of medium-term lending facilities (MLF) and targeted medium-term lending facilities

If the medium-term lending facility is combined with the specific entity conditional triggering management method, it can accurately determine the money supply. By adjusting the cost of medium-term financing to financial institutions, the balance sheets of financial institutions and market expectations will be influenced, and they will be guided to provide low-cost funds to the real economy sector in line with the national policy orientation. In this way, relevant entities will better implement structural monetary policies, reduce monetary idleness, make macro regulation more flexible, targeted and effective, thence enhance the ability of the financial sector to serve the real economy.

#### 5.3.2 Targeted implementation of pledged supplementary lending (PSL) to serve key sectors of the national economy

The purpose of creating Pledged Supplementary Lending is to provide financial institutions with long-term, stable, appropriate cost sources of funds and long-term large-scale financing in order to support the development of key areas, weak links and social undertakings of the national economy. If combined with the directional use of e-CNY [77], it can ensure that the transfer of funds can only occur when they are verified to meet the use rules. Besides, the directional use of e-CNY can be managed, so as to ensure the accuracy of the implementation of PSL and better serve economic development.

### 5.4 E-CNY could bring new monetary policy tools

#### 5.4.1 A feasible way of “helicopter money”

After the 2008 financial crisis, some scholars proposed that the use of "helicopter money" can make the distribution of wealth more equitable, and the "helicopter money" policy will be more direct and effective than quantitative easing and conventional monetary policy to stimulate the economy [78, 79]. However, in practice, the central bank has not found the best way to "spray helicopter money". The emergence of CBDC will provide a feasible way for central banks to “helicopter money” through digital currency accounts, thereby enabling the effective implementation of this monetary policy tool [57]. Although the People’s Bank of China does not currently have such a plan, it can still be used as a back-up policy.

#### 5.4.2 Break the effective lower bound of zero interest rate by means of wallet custody fee

Although the People’s Bank of China is not facing the dilemma of negative interest rate policy at present, some countries have to lower the benchmark interest rate or even set it close to zero in the face of economic downturn to stimulate economic recovery and fight deflation in the future, and the lower bound of deposit interest rate also hinders the effect of monetary policy transmission. The central bank can break the constraint of the zero lower bound on interest rates and expand monetary policy space by levying wallet storage fees on CBDC or adjusting the interest rates of the CBDC. By these means e-CNY could be modified to weaken the zero lower bound, although it is not currently designed.

### 5.5 The e-CNY will have an overall positive impact on the ultimate targets of monetary policy

According to Article 3 of the Law of the People’s Republic of China on the People’s Bank of China, the objective of China’s monetary policy is to maintain the stability of the value of the currency and thereby promote economic growth.

#### 5.5.1 Promote economic growth

At present, digital economy is becoming a new blue ocean and new driving force for China’s economic development. The convenience, security and stability of e-CNY are also consistent with the rapidity and efficiency pursued by digital economy, which will promote the rapid development of China’s digital economy. In addition, the issuance of e-CNY can prevent financial systemic risks, reduce the moral hazard of commercial banks, non-bank payment institutions and other private institutions, significantly reduce the space for illegal activities, and make economic development more stable.

Due to the limited scope of e-CNY pilot and the small amount of published data, some scholars have simulated the economic impact of e-CNY. They found that the impact on banking system and financial structure is controllable. In the long run, it will help increase economic output [80–82].

#### 5.5.2 A certain risk of inflation

The issuance of e-CNY will bring about the expansion of money velocity and money multiplier, as a result, it is more difficult for the central bank to achieve the goal of price stability in the short term. In addition, the issuance cost of digital currency is lower, which requires the central bank to be more disciplined in issuing currency to avoid over-issuance.

On the other hand, with the help of big data and artificial intelligence and other technologies, the central bank can realize the real-time monitoring of the price level, adjust the speed of money supply with the help of information advantages, thus timely adjust money supply, and maintain the stability of prices, minimize the possible inflationary effects.

#### 5.5.3 More job opportunities to help achieve full employment

The issuance of e-CNY requires the participation of operators, hardware manufacturers, network maintenance departments and other parties, which will also bring new jobs to the society.

#### 5.5.4 Enhance exchange rate stability

After the issuance of e-CNY, it can realize the tracking of capital flow, facilitate the understanding of the scale of currency spillover, and then reduce the behavior of capital withdrawal using CNY, and enhance the stability of exchange rate. In addition, e-CNY will also be conducive to improving the level of anti-money laundering, anti-terrorist financing and anti-tax evasion, and expanding the two-way opening of the financial industry. Besides, the efficient clearing and settlement method of e-CNY will reduce transaction costs and improve payment efficiency, which is more conducive to the operation of cross-border payment system. The 1:1 free exchange with CNY can connect with the existing monetary system of various sovereign countries in the world, and further promote the internationalization of CNY through cross-border payment.

### 5.6. There may exist some possible negative effects of e-CNY on monetary policy

#### 5.6.1 Financial Disintermediation at crisis time

As the safest asset, e-CNY could exacerbate bank runs in times of crisis. Residents and companies can easily convert deposits into e-CNY, which may cause financial disintermediation and amplify financial volatility [26, 56, 83]. In particular, when systemic risks emerge, CBDC will serve as a channel for the public to quickly switch to safe assets.

#### 5.6.2 Limited substitution to other payment instruments

Since e-CNY does not bear interest, this may reduce the attractiveness of it, because people may have to give up the interest benefits brought by deposits and other assets, which is an additional opportunity cost [84].

Although the "dual offline" function of e-CNY enables the users to complete payments without a network, it still depends on the integrity of electricity and payment equipment, and cannot be used in the event of a power outage or in the event of a natural disaster. Besides, some people rely more on cash in times of crisis for psychological reasons. Therefore, on some occasions, cash is more advantageous. And if e-CNY is not widely used, it will be difficult to make full use of its economic advantage on monetary policy.

#### 5.6.3 Competing with private money

According to the latest statistics from the Coinmarketcap website, there are more than 10,000 types of digital currencies, and the total market value of these digital currencies exceeds 2.5 trillion. They have a certain competitive relationship with CBDCs including e-CNY, and impairing the ability of the central bank to conduct monetary policy as a monopoly.

## 6. Conclusion and enlightenment

Research shows that the issuance of e-CNY will have a significant impact on monetary policy: (1) From the perspective of money demand, it is an optimization of the existing payment system, which will have a positive impact on the implementation of monetary policy, so as to change the structure of money demand and improve the money velocity. (2) From the perspective of money supply, E-CNY issuance will increase the volatility and expansion effect of money multiplier to a certain extent, and make the intermediate target of monetary policy more controllable and reliable by better controlling the total money supply, shortening the time lag of interest rate transmission and effectively monitoring the deposit fund of deposit account. (3) From the perspective of monetary policy tools, E-CNY can improve the transmission effect of existing monetary policy tools and dredge the transmission channel of monetary policy by optimizing the reserve requirements policy, improving the flexibility of open market operation and giving better play to the advantage of the interest rate corridor of standing lending facility. Through the combination with structural monetary policy tools, the central bank will achieve targeted money supply, better implement medium-term lending facilities, precise implementation of mortgage supplementary loans. E-CNY could also bring new monetary policy tools, provide a way of “helicopter money”. (4) The issuance of e-CNY will have a positive impact on the ultimate targets of monetary policy. Therefore, in the context of the gradual circulation of e-CNY and its impact on monetary policies, this paper puts forward the following policy suggestions:

### 6.1 Further strengthen the theoretical research of e-CNY and make adequate plans

Before the nationwide issuance of e-CNY, it is necessary to further strengthen the theoretical research of e-CNY, and further explore the mechanism of its effect on money supply and demand, money multiplier and transmission efficiency of monetary policy. In addition, mathematical models can be built to reveal the process and results of the influence of e-CNY on monetary policy, and make sufficient contingency plans to deal with negative effects, so as to build a policy framework and theoretical basis for comprehensive promotion.

### 6.2 Fully use the e-CNY pilot data to test the impact of e-CNY on monetary policy

In the pilot areas of e-CNY, statistical monitoring and penetrating management should be strengthened with big data means, theoretical model construction, and empirical research should be carried out with relevant data, for example, the volatility and expansion effect of currency multiplier should be studied, and the possible impact of e-CNY on economic society and monetary policy should also be monitored. In addition, comparative studies should be carried out between pilot and non-pilot areas to summarize the advantages of e-CNY issuance, verify the reliability of e-CNY and improve the effectiveness of monetary policy, and reveal the process and results of the influence of e-CNY on monetary policy.

### 6.3 Strengthen the innovation of monetary policy tools and build a monetary policy system under the framework of e-CNY

The e-CNY is programmed with a variety of technical features, which can better regulate the total amount of money supply and monitor the whereabouts of money in real time, contributing to the better implementation of monetary policy. With the continuous maturity of e-CNY research and development, it is necessary to strengthen the innovation of monetary policy tools in the future, organically combine the advantages of e-CNY with monetary policy, and build a monetary policy system under the background of e-CNY framework, so as to make the transmission effect of monetary policy better, more time-effective and more accurate, and serve the development of real economy.

Due to the limited scope and transaction scale of the current pilot of e-CNY, there is not enough data to support empirical analysis. Therefore, this article only deduces the possible impact of e-CNY on monetary policy based on the pilot and released patents. In the future, with the expansion of the e-CNY pilot, more empirical analysis is worthy of further study.

## Supporting information

S1 Dataset Dataset of money supply. This file is the data source for Figs 2, 4, 5 in the text.
(XLSX)

## References

[1] Ali R, Barrdear J, Clews R, et al. The economics of digital currencies. Bank of England Quarterly Bulletin, 2014; Q3.

[2] Gans J S, Halaburda H. 9. Some Economics of Private Digital Currency. University of Chicago Press, 2015.

[3] Chuen David Lee Kuo, ed. Handbook of digital currency: Bitcoin, innovation, financial instruments, and big data[M]. Academic Press, 2015.

[4] Berentsen A., & Schär F. A short introduction to the world of cryptocurrencies. Federal Reserve Bank of St. Louis Research Paper Series, Review, 2018; 100(1), 1–16.

[5] Carney M. The future of money. In Scottish Economics Conference. Edinburgh: Edinburgh University. 2018; March.

[6] Adrian T, Mancini-Griffoli T. The rise of digital money. Annual Review of Financial Economics, 2019; 13.

[7] Mikhaylov A Y. Development of Friedrich von Hayekʼs theory of private money and economic implications for digital currencies. Terra Economicus, 2021; 53–62.

[8] Bech M L, Garratt R. Central bank cryptocurrencies. BIS Quarterly Review September, 2017.

[9] Grinberg R. Bitcoin: An innovative alternative digital currency. Hastings Sci. & Tech. LJ, 2012; 4: 159.

[10] Yermack D. Is Bitcoin a real currency? An economic appraisal. In Handbook of digital currency. Academic Press, 2015; 31–43.

[11] Tschorsch F, Scheuermann B. Bitcoin and beyond: A technical survey on decentralized digital currencies. IEEE Communications Surveys & Tutorials, 2016; 18(3): 2084–2123.

[12] Ruppelt M.N., et al., Bitcoin: A proposal of digital currency. Revista Technologia Sociedade, 2019; 15(38): p. 274–302.

[13] Zheng B, Zhu L, Shen M, et al. Identifying the vulnerabilities of bitcoin anonymous mechanism based on address clustering. Science China Information Sciences, 2020; 63(3): 1–15.

[14] Jin H, Xiao J. Towards trustworthy blockchain systems in the era of “Internet of value”: development, challenges, and future trends. Science China Information Sciences, 2022; 65(5): 1–11.

[15] Fradera F. Conference Report on ‘Digital Revolution: Data Protection, Artificial Intelligence, Smart Products, BlockchainTechnology and Virtual Currencies. Challenges for Law in Practice’. European Review of Private Law, 2018; 26(5).

[16] Wu Y, Fan H, Wang X, et al. A regulated digital currency. Science China Information Sciences, 2019; 62(3): 32109.

[17] Lu W, Wu L, Zhao R, et al. Blockchain technology for governmental supervision of construction work: learning from digital currency electronic payment systems. Journal of Construction Engineering and Management, 2021; 147(10): 04021122.

[18] Working Group on E-CNY Research and Development of the People’s Bank of China. Progress of Research & Development of E-CNY in China. 2021; July.

[19] Boar C, Wehrli A. Ready, steady, go? -Results of the third BIS survey on central bank digital currency. 2021.

[20] Yao Q. A systematic framework to understand central bank digital currency. Science China Information Sciences, 2018; 61(3): 1–8.

[21] Yao Q. Central Bank Digital Currency: optimization of the currency system and its issuance design. China Economic Journal, 2019; 12(1): 1–15.

[22] Fung B S C, Halaburda H. Central bank digital currencies: a framework for assessing why and how. Available at SSRN 2994052, 2016.

[23] Broadbent B. Central Bank and Digital Currencies. Speech at the London School of Economics, 2016; 2. www.bankofengland.co.uk/publications/Pages/speeches/default.aspx

[24] Bjerg O., & Nielsen R. H. Who Should Make Kroner? -A Review of Danmarks Nationalbank’s Analysis of CBDC. CBS Working Paper. 2018.

[25] Huber J. Digital currency. Design principles to support a shift from bankmoney to central bank digital currency. Real-world Economics Review, 2019; 76.

[26] Armelius H, Guibourg G, Johansson S, et al. E-krona design models: pros, cons and trade-offs. Sveriges Riksbank Economic Review, 2020; 2: 80–96.

[27] Fernández-Villaverde J, Sanches D, Schilling L, et al. Central bank digital currency: Central banking for all?. Review of Economic Dynamics, 2021; 41: 225–242.

[28] Supervision Basel Committe For Banking, “Risk Management for Electronic Banking and Electronic Money Activities.” Basle Committee on Banking Supervision, 1998; 35.

[29] Fälth Magnus. Electronic Money and the Future of Central Banks. economic commentary, 2002.

[30] Slovinec Marko. Digital Money and Monetary Policy. BIATEC, 2006; 14: 3.

[31] Skeie D. R. “Banking with Nominal Deposits and Inside Money.” Journal of Financial Intermediation, 2008; 17(4): 562–584.

[32] Fujiki H, Tanaka M. Currency demand, new technology, and the adoption of electronic money: Micro evidence from Japan. Economics Letters, 2014; 125(1):5–8.

[33] Bank for International Settlements. Central bank digital currencies. BIS Committee on Payments and Market Infrastructures, Market Committee, March,2018.

[34] Sinelnikova-Muryleva E V. Central bank digital currencies: Potential risks and benefits. Voprosy Ekonomiki, 2020; (4): 147–159.

[35] Cullen J. “Economically inefficient and legally untenable”: constitutional limitations on the introduction of central bank digital currencies in the EU. Journal of Banking Regulation, 2021; 1–11.

[36] Wu T. and Chen J. A study of the economic impact of central bank digital currency under global competition. China Economic Journal, 2021; 14(1): 78–101.

[37] Lee D K C, Yan L, Wang Y. A global perspective on central bank digital currency. China Economic Journal, 2021; 14(1): 52–66.

[38] Kochergin D. Central Banks Digital Currencies: World Experience. Mirovaia ekonomika i mezhdunarodnye otnosheniia, 2021; 65(5): 68–77.

[39] Kirkby R. Cryptocurrencies and digital fiat currencies. Australian Economic Review, 2018; 51(4): 527–539.

[40] Bindseil U. Central bank digital currency: Financial system implications and control. International Journal of Political Economy, 2019; 48(4): 303–335.

[41] Cukierman A. Reflections on welfare and political economy aspects of a central bank digital currency. The Manchester School, 2020; 88: 114–125.

[42] Belke A. and Beretta E. From cash to central bank digital currencies and cryptocurrencies: a balancing act between modernity and monetary stability, Journal of Economic Studies, 2020; 7(4): 911–938.

[43] Tarik Hansen, and Delak Katya. Security Considerations for a Central Bank Digital Currency, FEDS Notes. Washington: Board of Governors of the Federal Reserve System, 2022; February 03, doi: 10.17016/2380-7172.2970

[44] Samudrala R.S., Yerchuru S.K. Central bank digital currency: risks, challenges and design considerations for India. CSIT 9, 2021; 245–249. doi: 10.1007/s40012-021-00344-5

[45] Engert W., & Fung B. S. C. Central bank digital currency: Motivations and implications (No. 2017–16). Bank of Canada Staff Discussion Paper. 2017.

[46] Pfister C. Monetary policy and digital currencies: Much ado about nothing? Bank of France (No. 642). Working Papers. 2017.

[47] Meaning J., Dyson B., Barker J., & Clayton E. Broadening narrow money: monetary policy with a central bank digital currency. Social Science Electronic Publishing. 2018.

[48] Brunnermeier M K, Niepelt D. On the equivalence of private and public money. Journal of Monetary Economics, 2019; 106: 27–41.

[49] Andolfatto D. Assessing the impact of central bank digital currency on private banks. The Economic Journal, 2021; 131(634): 525–540.

[50] Davoodalhosseini S M. Central bank digital currency and monetary policy. Journal of Economic Dynamics and Control, 2021; 104150.

[51] Monnet C, Petursdottir A, Rojas-Breu M. Central Bank Account For All: Efficiency and Stability. Mimeo, 2019.

[52] Barrdear J, Kumhof M. The macroeconomics of central bank digital currencies. Journal of Economic Dynamics and Control, 2021; 104148.

[53] Rahman A J. Deflationary policy under digital and fiat currency competition. Research in economics, 2018; 72(2): 171–180.

[54] Bjerg O. Designing new money-the policy trilemma of central bank digital currency. CBS Working Paper. 2017.

[55] Raskin M., & Yermack D. Digital currencies, decentralized ledgers and the future of central banking. In Research handbook on central banking. Edward Elgar Publishing. 2018.

[56] Keister T., & Sanches D. R. Should central banks issue digital currency? http://www.toddkeister.net/pdf/KS_CBDC.pdf. 2019.

[57] Bordo M. D., & Levin A. T. Central bank digital currency and the future of monetary policy (No. w23711). National Bureau of Economic Research. 2017.

[58] Bank Norges. Central bank digital currencies. Norges Bank Paper No 1/2018.

[59] Riksbank Sveriges. Riksbank e-krona project. Report 2. 2018.

[60] Harrison R., & Thomas R. Monetary financing with interest-bearing money. Bank of England Staff Working Paper No. 785. 2019.

[61] Gürtler K, Nielsen S, Rasmussen K, et al. Central bank digital currency in Denmark. Danmarks Nationalbank Analysis, 28. 2017.

[62] Wadsworth A. The pros and cons of issuing a central bank digital currency. Reserve Bank of New Zealand Bulletin, 2018; 81, 1–21.

[63] Yanagawa N., & Yamaoka H. Digital Innovation, Data Revolution and Central Bank Digital Currency (No. 19-E-2). Bank of Japan. 2019.

[64] Dong M., & Xiao S. X. Central Bank Digital Currency: A Corporate Finance Perspective. Available at SSRN 3911132. 2021.

[65] Priyatama Abednego, and Apriansah Apriansah. Correlation between electronic money and the velocity of money. Global Management Conference. Bali, 2010.

[66] Chen Y. The impact of digital payments on the velocity of money in the Chinese market. Universidade de Lisboa, Doctoral dissertation. 2021.

[67] Liu X., Liu Q. Study on the Influence of Internet Payment on the Velocity of Money Circulation in China. In: Hassanien A.E., Xu Y., Zhao Z., Mohammed S., Fan Z. (eds) Business Intelligence and Information Technology. BIIT 2021. Lecture Notes on Data Engineering and Communications Technologies, vol 107. Springer, Cham. 2022.

[68] Zhou L., Chen S., Xue B. Influence of Central Bank Digital Currency on Monetary Policy. The Chinese Banker.2019;vol 10: 55–58. (in Chinese)

[69] Li-juan G. U. O., and Pei-long S. H. E. N. Central Bank Digital Currency, Bank Stability and Economic Growth: Theory and Prediction. Commercial Research 2020; 62(9): 100.

[70] Kiriakova N I. Digital Currencies of the Central Bank as a Means of Strengthening the Monetization of the Russian Economy. 2nd International Scientific and Practical Conference “Modern Management Trends and the Digital Economy: from Regional Development to Global Economic Growth”(MTDE 2020). Atlantis Press, 2020; 1155–1159.

[71] Digital Currency Research Institute of People’s Bank of China. The Time-point Conditional Triggering Management Method of Digital Currency. China. Patent. 201810252498.9. 2018-3-26.

[72] Digital Currency Research Institute of People’s Bank of China. The Specific Entity Conditional Triggering Management Method of Digital Currency. China. Patent. 201810251582.9. 2018-3-26.

[73] Digital Currency Research Institute of People’s Bank of China. The Loan Interest Rate Conditional Triggering Management Method of Digital Currency. China. Patent. 201810252499.3. 2018-3-26.

[74] Digital Currency Research Institute of People’s Bank of China. The Economic State Conditional Triggering Management Method of Digital Currency. China. Patent. 201810252512.5. 2018-3-26.

[75] Fang X., Huang S. Digital Currency and China’s Monetary Policy Transformation. Academic Forum. 2020; 2:91–101. (in Chinese)

[76] Xie X., Feng S. Theoretical Research on the Impact of Central Bank Digital Currency on Chinese Monetary Policy. Economist. 2019; 9: 54–63. (in Chinese)

[77] Digital Currency Research Institute of People’s Bank of China. Method and Device for Targeted Use of Digital Currency. China. Patent. 201710496964.3. 2017-6-26.

[78] Bernake B. Revisiting The Helicopter Speech. Speech Given at National Economists Club, Washington, D.C., November, 2002.

[79] Di Giorgio G, Traficante G. Fiscal shocks and helicopter money in open economy. Economic Modelling. 2018; 74: 77–87.

[80] Yao Q. Analysis of the Economic Effects of Central Bank Digital Currency: Theory and Empirical Studies. Financial Theory & Policy. 2019; 1:16–27. (in Chinese)

[81] Lan T., Pang C., Xiao J., Zhang X. Central Bank Digital Currency: Forward Triggering and Monetary Policy Transmission. South China Finance. 2021; 2: 38–52. (in Chinese)

[82] Xie X., Zhang Y., Feng S. Study of the Macroeconomic Effect of Central Bank Digital Currency. Finance & Trade Economics. 2020; 10:147–161. (in Chinese)

[83] Fan Y. Theoretical Basis and Architecture Choice of China’s Legal Digital Currency. China Finance, 2016; (17): 10–12. (in Chinese)

[84] Borgonovo E, Cillo A, Caselli S, et al. Between Cash, Deposit and Bitcoin: Would We Like a Central Bank Digital Currency? Money Demand and Experimental Economics. Money Demand and Experimental Economics. BAFFI CAREFIN Centre Research Paper, 2018; 2018–75.

